# Structural basis for the recognition of guide RNA and target DNA heteroduplex by Argonaute

**DOI:** 10.1038/ncomms11846

**Published:** 2016-06-21

**Authors:** Tomohiro Miyoshi, Kosuke Ito, Ryo Murakami, Toshio Uchiumi

**Affiliations:** 1 Center for Transdisciplinary Research, Niigata University, 8050 Ikarashi 2-no-cho, Nishi-ku, Niigata 950-2181, Japan; 2 Department of Biology, Faculty of Science, Niigata University, 8050 Ikarashi 2-no-cho, Nishi-ku, Niigata 950-2181, Japan

## Abstract

Argonaute proteins are key players in the gene silencing mechanisms mediated by small nucleic acids in all domains of life from bacteria to eukaryotes. However, little is known about the Argonaute protein that recognizes guide RNA/target DNA. Here, we determine the 2 Å crystal structure of *Rhodobacter sphaeroides* Argonaute (*Rs*Ago) in a complex with 18-nucleotide guide RNA and its complementary target DNA. The heteroduplex maintains Watson–Crick base-pairing even in the 3′-region of the guide RNA between the N-terminal and PIWI domains, suggesting a recognition mode by *Rs*Ago for stable interaction with the target strand. In addition, the MID/PIWI interface of *Rs*Ago has a system that specifically recognizes the 5′ base-U of the guide RNA, and the duplex-recognition loop of the PAZ domain is important for the DNA silencing activity. Furthermore, we show that Argonaute discriminates the nucleic acid type (RNA/DNA) by recognition of the duplex structure of the seed region.

## Abstract (web summary)

Argonaute proteins are important in the silencing machinery with some regulatory RNAs. Here, the authors solve the structure of an argonaute protein in complex with both the guide RNA and target DNA and propose a mechanism for their recognition.

## Graphical abstract

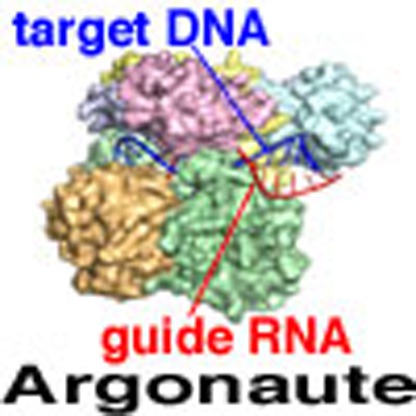

Argonaute-family proteins have crucial roles in gene regulation in collaboration with small guide strands, which recognize complementary sequences in the target^1^. This recognition occurs mainly through the seed region, a 2–8 nucleotide segment of the guide strands^2^,^3^,^4^. The crystal structures of full-length Argonaute proteins have been investigated to help understand the mechanism of their action; the first structures were reported in the prokaryotes *Pyrococcus furiosus* and *Aquifex aeolicus*^5^,^6^. Argonaute proteins belong to the PIWI protein superfamily, and contain a PIWI (P element-induced wimpy testis) domain, a MID (middle) domain, a PAZ (Piwi–Argonaute–Zwille) domain and an N-terminal domain. The proteins form a bilobal scaffold composed of MID–PIWI and N–PAZ lobes connected with linkers. This bilobal structure binds to guide and target molecules along a nucleic acid-binding channel^7^,^8^,^9^,^10^,^11^,^12^,^13^,^14^,^15^,^16^. The PIWI domain of Argonaute is a structural homologue of the RNase H catalytic domain, and contributes to endonuclease (slicing) activity for the target strand^5^,^17^. However, the slicing activity is not a property of all Argonaute proteins^17^,^18^,^19^. The MID domain consists of a Rossmann-like fold and interacts directly with the 5′-end of the guide strand by recognition of the phosphorylated first nucleotide and base features^20^,^21^. The PAZ domain contains a hydrophobic pocket of an oligosaccharide-binding-like fold and is involved in binding of a single oligonucleotide; the PAZ domain associates with the 3′-end of the guide strand^22^,^23^,^24^,^25^,^26^. This domain is required to unwind the small RNA duplex during RNA-induced silencing complex formation in non-cleavage Argonautes^27^ and to regulate slicer activity^28^. The N-terminal domain of Argonaute is the least well characterized of the four domains. Crystallographic analysis of the Argonaute complex in *Thermus thermophilus* (*Tt*Ago) showed that the N-terminal domain prevents extension of base pairing of the guide and target strands at the 3′-region of the guide strand^9^,^16^. In addition, biochemical studies of human Argonaute (*Hs*Ago) showed that the N-terminal domain facilitates the unwinding of the guide/target duplex during RNA-induced silencing complex assembly^29^.

Argonaute proteins are evolutionarily diverse^30^. In eukaryotes, Argonaute proteins are key players in the small ‘guide RNA'-mediated silencing systems for ‘target RNA', such as RNA interference, microRNA gene silencing and piRNA transposon silencing^31^. In contrast, prokaryotic Argonaute proteins from *T. thermophilus* and *P. furiosus* associate with DNAs as guide and target strands, and interfere with the propagation of foreign DNA^32^,^33^,^34^. This latter system is also called DNA-guided DNA interference^32^. A previous *in vitro* study showed that *Tt*Ago could be co-purified with 13–25 nucleotide (nt) single strand DNAs composed of plasmid sequences with a deoxycytidine at the 5′-end^32^. The *Tt*Ago complex with guide DNA recognizes an AT-rich sequence element in the target DNA, and cleaves plasmid and long double-stranded DNA by nicking two strands^32^. Studies with Argonaute proteins from *A. aeolicus*^6^, *P. furiosus*^33^ and *Methanocaldococcus jannaschii*^35^, and with Piwi proteins, which are composed of only the MID and PIWI domains, of *Archaeoglobus fulgigus*^36^ showed that these have a pronounced propensity to interact with DNA as the guide strand. In addition, these prokaryotic proteins containing guide DNA are able to associate with DNA (or potentially RNA) as the target strand^6^,^8^,^33^,^35^,^36^.

The slicer-deficient Argonaute protein from *R. sphaeroides* (*Rs*Ago) was co-purified with 5′ U-rich RNAs (15–19 nt) and DNAs (22–24 nt)^37^. However, the respective roles of RNA and DNA as guide/target strands are not clear in *Rs*Ago. It has been suggested that the *Rs*Ago complexes block loading of RNA polymerase and transcriptional elongation, leading to repression of transcription from target DNA in *R. sphaeroides* cells^37^. Furthermore, the complexes also inhibit propagation and promote degradation of plasmid DNA in a heterologous expression system using *Escherichia coli* cells^37^. The present study resolves these uncertainties over the mechanism of Argonaute action using a combination of biochemical, structural and mutagenesis analyses. Our biochemical analysis shows that *Rs*Ago specifically interacts with RNA and DNA as the guide and target strands, respectively, suggesting that *Rs*Ago has a key role in a unique silencing system, that is, RNA-guided DNA silencing. We determine the crystal structure of *Rs*Ago in complex with 18-base guide RNA and 18-base target DNA at 2 Å resolution. Our structural evidence reveals that the N-terminal domain of *Rs*Ago allows base-pairing propagation at the 3′ side of the guide strand in contrast to *Tt*Ago, in which the duplex is unwound by the N-terminal domain. In a comprehensive mutagenesis study of the amino acids interacting with the heteroduplex in *Rs*Ago, we identify many of the functionally important regions in *Rs*Ago, for example, the MID and N-terminal domains, which contribute to binding to the guide RNA and the target DNA, respectively, and the PAZ loop (but not the 3′-end-binding pocket of PAZ domain), which has a role in DNA silencing. Through use of chimeric nucleic acids and docking simulation, we also demonstrate that the duplex structure of the seed region is important in the RNA/DNA heteroduplex for recognition by *Rs*Ago.

## Results

### Types of nucleic acid bound to *Rs*Ago

**Figure 1:**
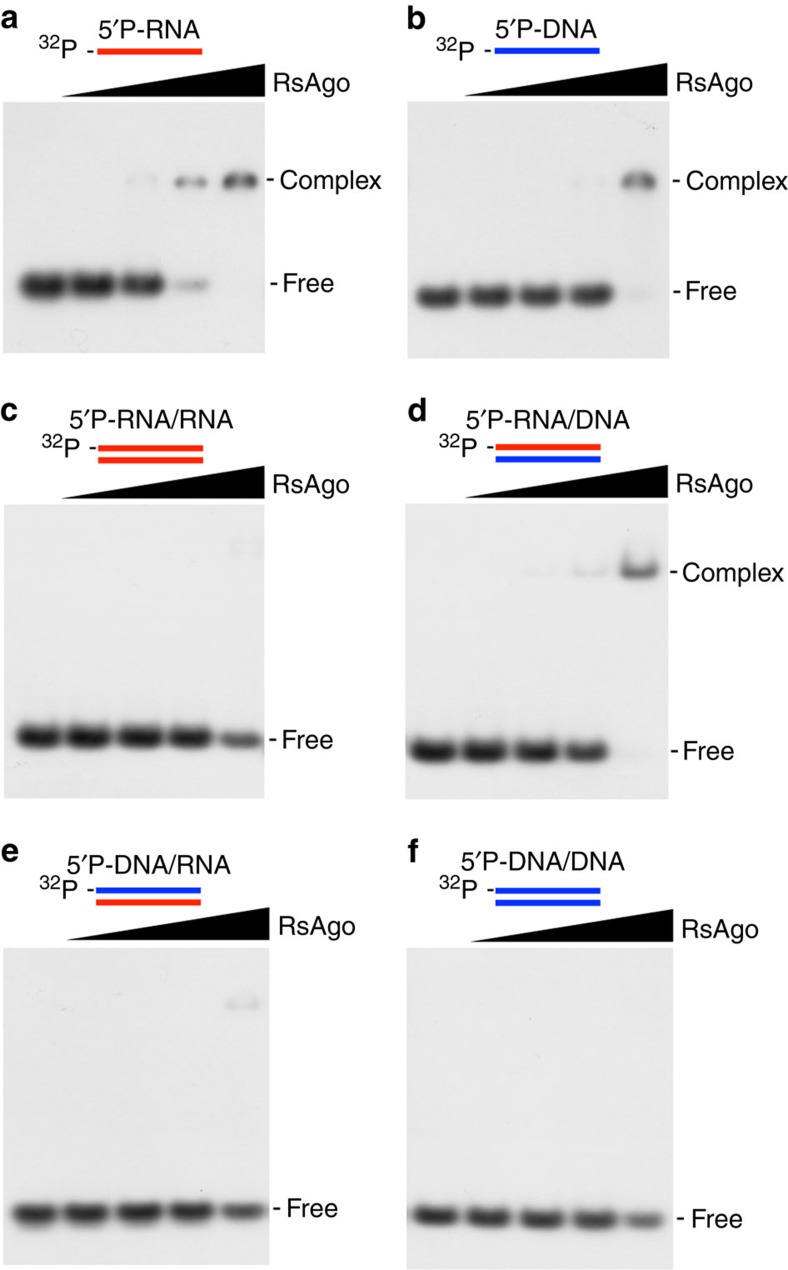
Nucleic acid types of 18-base guide and target strands bound to *Rs*Ago. (**a**,**b**) Binding affinities of 5′P-RNA (5′P-RNA) (**a**) and DNA (5′P-DNA) (**b**) to the WT recombinant *Rs*Ago protein were examined using an electrophoresis mobility shift assay. The oligonucleotide sequences of the ^32^P-labelled guide strands are shown in Supplementary Table 1. Single strand RNA and DNA (5 nM each) were incubated with various concentrations (0, 1, 10, 100 and 1000, nM) of WT *Rs*Ago. (**c–f**) Binding affinities of 5′P-RNA/non-phosphorylated RNA (5′P-RNA/RNA) (**c**), 5′P-RNA/non-phosphorylated DNA (5′P-RNA/DNA) (**d**), 5′P-DNA/non-phosphorylated RNA (5′P-DNA/RNA) (**e**), 5′P-DNA/non-phosphorylated DNA (5′P-DNA/DNA) (**f**) duplexes to the WT recombinant *Rs*Ago protein were examined using an electrophoresis mobility shift assay. The oligonucleotide sequences of the ^32^P-labelled guide and target strands are shown in Supplementary Table 1. The duplexes were incubated in a same way as **a**,**b**. The small RNA and DNA strands are shown as red and blue lines, respectively.

We selected Argonaute from *R. sphaeroides* (*Rs*Ago) to investigate the recognition mechanisms for the guide and target strands. The Argonaute protein of *R. sphaeroides* lacks slicer activity and thus permits the formation of Argonaute complexes containing the guide strand and the uncleaved target strand. A previous study reported that *Rs*Ago associates with a 15–19 nt RNA molecule and a 22–24 nt DNA molecule^37^. However, the nucleic acid types that act as the guide or target molecules for *Rs*Ago were not determined. To address this question, we first performed an electrophoretic mobility shift assay using 18-base single strand RNA and DNA. In this assay, 5′-phosphorylated (5′P) strands were used because 5′-phosphorylation is known to be required for the recognition of the guide strand by Argonaute proteins^36^,^38^. Our assay showed that the binding ability of *Rs*Ago to the 18-base single strand RNA was higher than to the 18-base single strand DNA (Fig. 1a,b and Supplementary Table 1). Measurement of the equilibrium dissociation constant (Kd) values by a fluorescence polarization assay indicated that the 5′P 18-base RNA bound 46-fold more tightly to *Rs*Ago than to the 5′P 18-base single strand DNA (Kd: 5′P-RNA, 0.91±0.06 nM; 5′P-DNA, 42.6±3.4 nM) (Table 1 and Supplementary Fig. 1a,b). This result is consistent with those of the electrophoretic mobility shift assay (Fig. 1a,b).

To examine the specificity of *Rs*Ago for the nucleic acid duplexes, we carried out similar electrophoretic mobility shift assays using four types of complementary 18-base double strands, namely, 5′P-RNA/RNA, 5′P-RNA/DNA, 5′P-DNA/RNA and 5′P-DNA/DNA. Formation of an *Rs*Ago–duplex complex and the significant disappearance of free strands were unambiguously observed only when 5′P-RNA and non-phosphorylated DNA were used (Fig. 1c–f and Supplementary Table 1). Considering that 5′-phosphorylation is necessary for the recognition of the guide strand^36^,^38^, these results suggested that *Rs*Ago specifically recognized RNA as the guide strand and DNA as the target strand. To confirm the specificity of *Rs*Ago for the guide and target strands, we carried out the electrophoretic mobility shift assays by sequentially adding the guide and target strands to *Rs*Ago (Supplementary Fig. 2). The results showed that the DNA strand only bound to *Rs*Ago that had been pre-incubated with 5′P-RNA. This observation further supports our interpretation that *Rs*Ago specifically recognizes RNA as the guide strand and DNA as the target strand.

### Overall structure of the *Rs*Ago–RNA/DNA ternary complex

**Figure 2:**
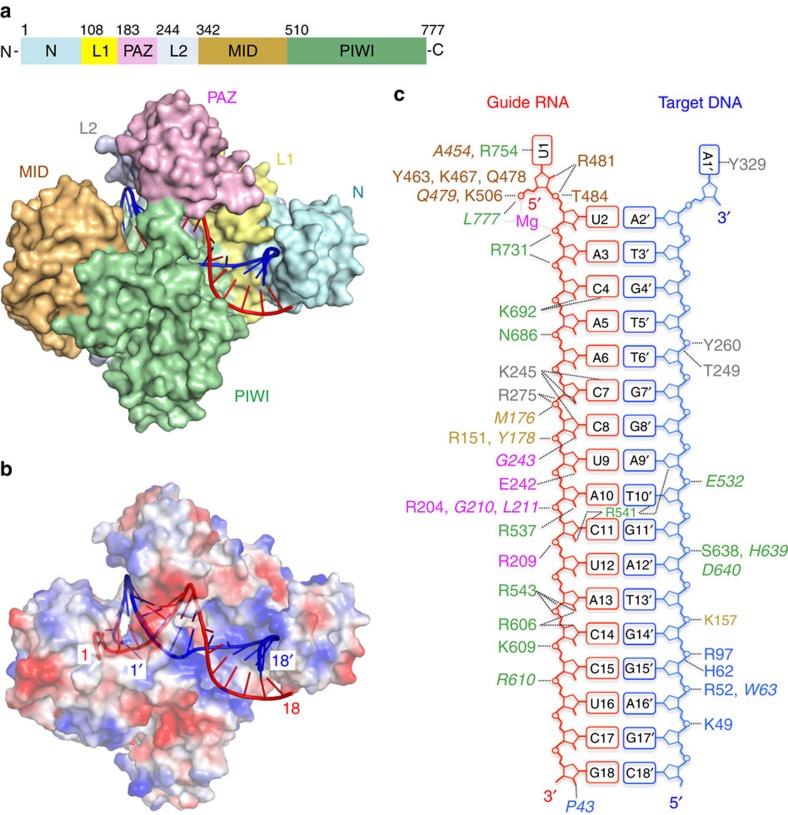
Crystal structure of *Rs*Ago bound to a 5′P 18-base guide RNA strand and 18-base target DNA strand. (**a**) View of the *Rs*Ago structure bound to a heteroduplex. The Ago protein is shown here in surface representation with colour-coded domains and linkers. (**b**) In this structure, the Ago protein is shown in an electrostatically colour-coded surface representation. The full lengths of the heteroduplexes in both structures could be traced and are shown in a cartoon representation coloured in red (guide RNA strand) and blue (target DNA strand). (**c**) Schematic diagram of intermolecular contacts between *Rs*Ago and guide RNA (1–18)/target DNA (1′–18′) heteroduplex. The sequence of the 5′P 18-base guide RNA strand is shown in red and that of 18-base target DNA strand is shown in blue. Dashed lines indicate hydrogen bonds between *Rs*Ago and guide RNA/target DNA heteroduplex. The colour-coding of the amino acid number is the same as in Fig. 2a. The residue numbers of the amino acids that interact with the duplex via side and main chains are indicated in normal and italic types, respectively.

On the basis of the results of the nucleic acid recognition assay (Fig. 1), we crystallized *Rs*Ago complexed with a hybrid duplex containing a 5′P 18-base guide RNA strand and an 18-base complementary DNA target strand, and solved the structure at 2 Å resolution using the single-wavelength anomalous dispersion method with SeMet-labelled proteins (Fig. 2a and Table 2). *Rs*Ago was composed of N-terminal, PAZ, MID and PIWI domains, and L1 and L2 linkers (Fig. 2a). Overall, the structure of *Rs*Ago was similar to other reported Argonaute proteins^39^. The fully solved heteroduplex is bound within a positively charged channel between N–PAZ-containing and MID–PIWI-containing lobes by an extensive hydrogen-bonding network (Fig. 2b,c and Supplementary Fig. 3). The nucleotide at the 5′-end of the guide RNA strand and the nucleotide at the 3′-end of the target DNA strand were unpaired in *Rs*Ago (Fig. 2b,c), as in previously determined Argonaute complexes^8^,^9^,^15^,^16^,^36^,^38^,^40^. Surprisingly, the 3′-region of the guide RNA strand was base-paired with the 5′-region of the target DNA strand; this behaviour has not been reported previously for other Argonaute complexes^8^,^9^,^16^. We discuss the implications of this structure in more detail below in conjunction with the results of biochemical analyses.

### MID/PIWI interface for guide RNA 5′-end recognition

**Figure 3:**
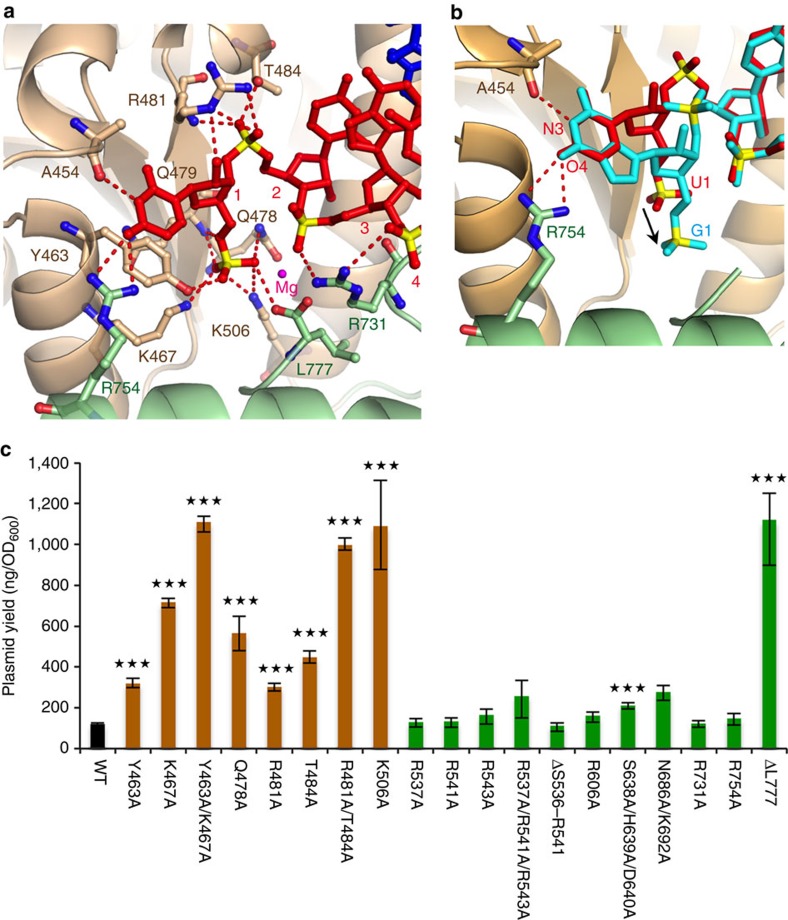
Interaction between the MID/PIWI interface and the 5′-end of guide RNA and the effects of mutation in *Rs*Ago. (**a**) Close-up view of the interaction between *Rs*Ago and the 5′-end of guide RNA. Hydrogen bonds are shown as dashed-red lines. The guide RNA (red) is shown in stick representation, with phosphorous atoms in yellow. Individual domains and linkers of the Argonaute protein are shown in cartoon representation, and with the same colour-coding as Fig. 2a. (**b**) Comparison of the 5′-phosphate position of nucleotide 1 (U and G) in the MID/PIWI interface of the *Rs*Ago ternary complex. Superposition of the guide RNA strand (red) with the 5′G-modelled guide RNA (cyan) in the *Rs*Ago ternary complex. Amino acid residues A454 and R754 are shown in stick representation. An arrow indicates the structural shift of the 5′-phosphate caused by mutation of the 5′ nucleotide from U to G. (**c**) Effect of *Rs*Ago mutations on plasmid DNA silencing. Plasmid DNAs, which express various *Rs*Ago variants in *E. coli* cells, were purified and the plasmid yields were quantified. Error bars represent s.d. values (*n*=3). ***P<0.001 compared with WT using the Student's *t*-test.

In the MID domain, the 5′-phosphate of the guide RNA strand forms hydrogen bonds with the side chains of Y463, K467, Q478, K506 and the main chains of Q479 (Fig. 3a). These amino acid residues, with the exception of Q479, are largely conserved in RNA-guided eukaryote Argonaute proteins as well as DNA-guided prokaryote Argonaute proteins (Supplementary Fig. 4)^36^, suggesting that the 5′-phosphate recognition mode is conserved irrespective of the nucleic acid type of the guide strand. In addition, the MID domain interacted with the 2′-hydroxy group of the nucleotide 1 and the phosphate group of nucleotide 2 of the guide RNA strand via R481 and T484 (Fig. 3a). Hydrogen bonds to the 2′-hydroxy group of the guide strand 5′-terminal nucleotide have also been observed in RNA-guided eukaryotic Argonaute proteins^11^,^13^,^14^, suggesting that this bond is important for the recognition of RNA as a guide strand.

To examine the contributions of the 5′-phosphate of the guide strand to the interaction with the MID domain, we measured the Kd values using a fluorescence polarization system. The elimination of the 5′-phosphate of the guide strand RNA severely impaired the binding activity to *Rs*Ago, as compared to the 5′P-RNA strand (Table 1 and Supplementary Fig. 1a,c). This result indicates that the 5′-phosphate of the guide strand RNA is an essential component for guide strand recognition by *Rs*Ago.

In addition to the interaction described above, the MID domain of *Rs*Ago forms base-specific interactions with the 5′-terminal uracil of the guide RNA strand. The aromatic residue Y463 stacked against the 5′-terminal uracil and the main chain of A454 formed a hydrogen bond with N3 of the uracil (Fig. 3a). Y463 is widely conserved across Argonaute proteins (Supplementary Fig. 4) and this conserved tyrosine has also been shown to stack against the 5′-terminal base of the guide strand in other Argonaute proteins^6^,^36^,^38^,^41^,^42^. However, other Argonaute proteins do not possess a residue corresponding to A454. These proteins contain a rigid loop corresponding to that containing A454; this rigid loop has been demonstrated to function as a critical determinant for the 5′-terminal nucleotide preference of the guide strand^20^,^21^. In addition to A454, the side chain of R754 of the PIWI domain also formed hydrogen bonds with O4 of the 5′-terminal uracil of the guide RNA strand (Fig. 3a). A similar hydrogen-bonding pattern with A454 and R754 was also possible if the 5′-terminal base was guanine. However, if the guanine formed hydrogen bonds with A454 and R754 like uracil, then the 5′-phosphate could no longer form the proper interaction with *Rs*Ago (Fig. 3b). Indeed, the measurement of Kd values using a fluorescence polarization system showed that the substitution of the 5′-nucleotide with guanosine severely impaired the binding activity to *Rs*Ago compared to 5′U-RNA (Kd: 5′U-RNA, 0.91±0.06 nM; 5′G-RNA, 181.3±13.3 nM) (Table 1 and Supplementary Fig. 1a,g). Furthermore, the substitution of the 5′-nucleotide with adenosine or cytosine also severely impaired the binding activity to *Rs*Ago (Kd: 5′A-RNA, 24.8±1.7 nM; 5′C-RNA, 67.0±3.5 nM) (Table 1 and Supplementary Fig. 1e,f). These structural and biochemical observations are in agreement with the fact that *Rs*Ago prefers 5′U-RNA^37^.

To investigate the functional role of the MID/PIWI interface of *Rs*Ago in the RNA-guided DNA silencing mechanism, we measured plasmid DNA silencing activity in *E. coli* cells^37^ using *Rs*Ago variants (Fig. 3c). On the basis of our present structural data, we selected the amino acid residues that interacted with nucleic acids via their side chains as the targets for mutation; initially, adjacent amino acid residues were substituted simultaneously. We confirmed that all mutants used in this study were expressed to the same level in *E. coli* cells (Supplementary Fig. 5). Mutants of the MID domain, Y463A/K467A, Q478A, R481A/T484A and K506A, strongly impaired plasmid DNA silencing activity (Fig. 3c). Subsequently, we measured the activity of single mutants, Y463A, K467A, R481A and T484A, and found that they also impaired the activity. To assess whether the reduced activity in the MID domain mutants was due to a decrease in the interaction with the guide strand, we examined the effects of mutations in the MID domain on the interaction with 5′P single strand RNA in a fluorescence polarization assay. The three most effective mutants from the plasmid DNA silencing assay (Y463A/K467A, R481A/T484A and K506A) were tested and all exhibited low affinity for the 5′P 18-base single strand RNA (Table 1 and Supplementary Fig. 1h–j). The effects of mutations of Y463, K467 and K506 on guide strand binding were consistent with the results from the PIWI protein from *A. fulgidus*^36^. In addition, the importance of R481 and T484 was demonstrated in the present experiment.

The present RNA-guided *Rs*Ago structure showed that a magnesium ion was coordinated by the first and third phosphates of the guide RNA strand and by the carboxyl group of L777 (Fig. 3a). The similar magnesium coordinate mode is also observed in other structures of DNA-guided Argonaute^7^,^8^,^9^,^16^, indicating the conservation of the magnesium coordination mode between DNA- and RNA-guided prokaryotic Argonautes. However, in eukaryotic Argonaute, the magnesium ion is replaced by a conserved lysine side-chain (K566 in *Hs*Ago2) (Supplementary Fig. 4)^10^,^11^,^12^,^13^,^14^,^15^. The plasmid DNA silencing assay showed that the C-terminal deletion variant ΔL777 strongly impaired plasmid DNA silencing activity (9.3-fold reduction) (Fig. 3c). We next examined the contributions of the carboxyl group of L777 and the magnesium ion to the interaction with 5′P guide RNA by measuring Kd values. The ΔL777 *Rs*Ago mutant exhibited low affinity for 5′P 18-base single strand RNA compared to wild-type *Rs*Ago (42-fold reduction) (Table 1 and Supplementary Fig. 1k). Removal of the magnesium by EDTA caused the binding ability of the 5′P guide strand RNA to wild-type *Rs*Ago to be reduced more than eightfold compared to that in the presence of magnesium (Table 1 and Supplementary Fig. 1d). These results indicate that the binding of 5′P guide strand RNA to *Rs*Ago is supported by the magnesium ion and the carboxyl group of L777 in the PIWI domain. In addition to the above-mentioned amino acid residues R754 and L777, the non-slicing PIWI domain of *Rs*Ago widely interacted with the heteroduplex. The details are described in the Supplementary Note (Supplementary Figs 3 and 6).

### Contribution of the PAZ domain to DNA silencing

**Figure 4:**
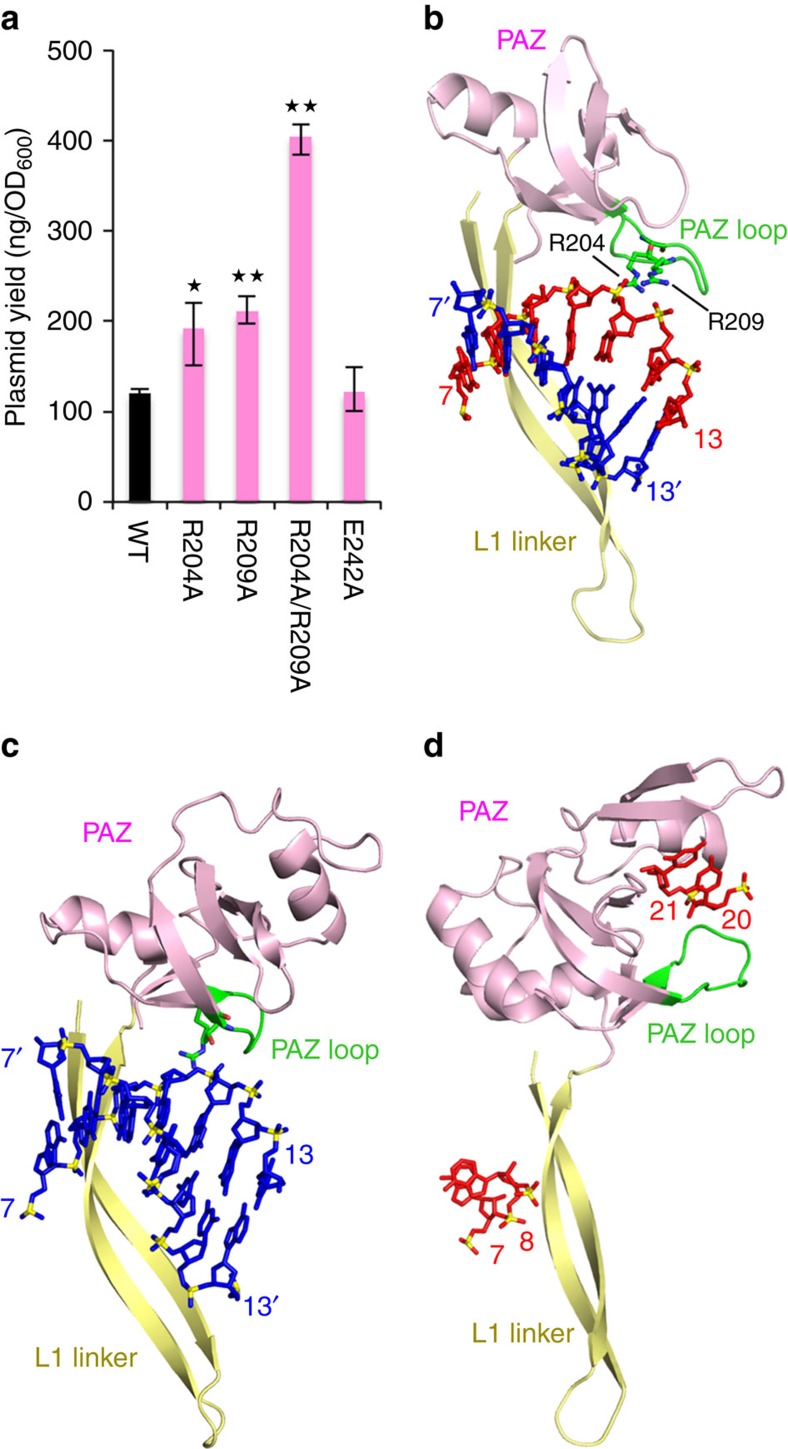
Functional interaction of the PAZ domain with the guide RNA strand. (**a**) Effect of the PAZ domain mutations on plasmid DNA silencing. Plasmid DNAs, which express various *Rs*Ago variants in *E. coli* cells, were purified and the plasmid yields were quantified. Error bars represent s.d. values (*n*=3). *P<0.05 and **P<0.01 compared with WT using the Student's *t*-test. (**b–d**) Structural conservation of the PAZ loops. The PAZ domains (pink), the PAZ loops (green) and L1 linkers (yellow) of *Rs*Ago (**b**), *Tt*Ago (**c**, PDB ID: 4NCB) and *Hs*Ago1 (**d**, PDB ID: 4KXT) are shown in cartoon representation. The guide RNA (red) and the target DNA (blue) bound to *Rs*Ago (**b**), the guide DNA (blue) and the target DNA (blue) bound to *Tt*Ago (**c**), and the guide RNA (red) bound to *Hs*Ago1 (**d**) are shown in stick representation with phosphorous atoms in yellow.

The PAZ domain of *Rs*Ago (R204, R209, G210, L211, E242 and G243) mainly interacted with the middle region (nucleotides 8, 9, 11 and 12) of the guide RNA strand in the heteroduplex (Fig. 2c and Supplementary Fig. 3d). To evaluate the functional significance of this interaction, we performed a plasmid DNA silencing assay using *Rs*Ago mutants of amino acid residues R204, R209 and E242, which interact with the heteroduplex via the side chains. The analysis showed that substitution of E242 with alanines had no marked effect on plasmid DNA silencing (Fig. 4a). However, the substitution of R204 or R209 with alanine, both of which are present in the same loop (which we named the PAZ loop, Fig. 4b), resulted in a significant reduction of the plasmid DNA silencing activity; the double mutation of these residues to alanines further reduced the activity (Fig. 4a). These results indicate the importance of the interaction between the PAZ loop and the guide RNA strand. In addition, the PAZ domain interacted with the guide RNA and target DNA together with L1 and L2 linkers. The details are described in the Supplementary Note (Supplementary Figs 3c,d and 7).

In the *Tt*Ago complex, the interaction of the PAZ loop with the guide strand is also observed^9^,^15^, although the nucleic acid type in the guide strand is different to that of *Rs*Ago (Fig. 4b,c). On the other hand, analogous interactions involving the PAZ loop have not been observed for eukaryotic Argonaute proteins, because the structure of the eukaryotic Argonaute-RNA/RNA duplex complex containing base pairs beyond the seed region is still unresolved (Fig. 4d). However, in view of the conservation of the interaction of the PAZ loop with the guide strand in *Rs*Ago and *Tt*Ago, and considering the importance of the PAZ loop for plasmid DNA silencing in *Rs*Ago, we suggest that the PAZ loop in the eukaryotic Argonaute protein will have an important role in the silencing activity.

In general, the PAZ domain of the Argonaute protein has two subdomains: an oligosaccharide-binding-fold like structure with one or two helices on one side; and an α-helix, a β-hairpin or loop structure followed by another α-helix (Supplementary Fig. 8a,b)^43^. These two subdomains are oriented to form a pocket (hereafter the PAZ pocket), and it has been demonstrated that the 3′-end of the guide strand is anchored within the PAZ pocket^22^,^23^,^25^,^26^. However, the PAZ domain of *Rs*Ago lacks the second subdomain, and therefore does not possess the PAZ pocket (Supplementary Fig. 8c). Thus, it seems that the binding mode of the 3′-end of the guide strand to the PAZ domain in *Rs*Ago is different from that of other Argonaute proteins, or that the 3′-end of the guide strand might not bind to the PAZ domain in *Rs*Ago. Further experiments are needed to clarify this point.

### Functional N-terminal domain for target strand association

**Figure 5:**
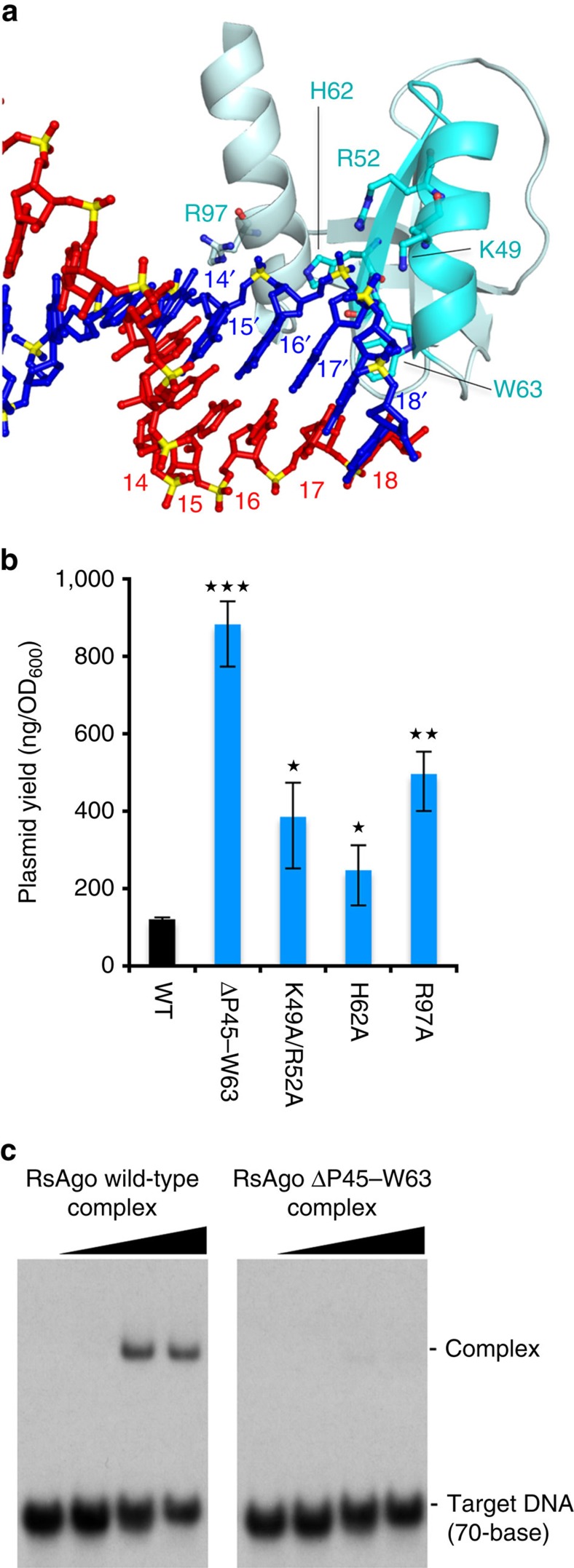
The N-terminal domain essential for the binding of the target DNA strand. (**a**) The 18-base guide RNA (red) and 18-base target DNA (blue) heteroduplex and the N-terminal domain (light blue) of *Rs*Ago. Amino acid residues that interact with the target strand are shown in stick representation. Residues P45–W63 are shown in cyan. (**b**) Effect of the N-terminal domain mutations on plasmid DNA silencing. Plasmid DNAs, which express various *Rs*Ago variants in *E. coli* cells, were purified and the plasmid yields were quantified. Error bars represent s.d. values (*n*=3). *P<0.05, **P<0.01 and ***P<0.001 compared with WT using the Student's *t*-test. (**c**) Electrophoresis mobility shift assay of the binding of the *Rs*Ago complex containing the guide RNA to the target DNA strand. The ^32^P-labelled 70-base target DNA (5 nM) was incubated with various concentrations (0, 1, 10 and 100 nM) of *Rs*Ago wild-type (left panel) or the ΔP45–W63 mutant (right panel) complex containing the 5′P 18-base guide RNA strand (Supplementary Table 1).

Our crystal structure of the *Rs*Ago complex showed that the 5′-region (nucleotides 14′–18′) of the target DNA strand in the heteroduplex was located on the positively charged surface of the N-terminal domain (Fig. 2b). On this surface, the target DNA strand (nucleotides 14′–16′) formed hydrogen bonds with the side chains of K49, R52, H62 and R97, and the main chain of W63 (Fig. 5a and Supplementary Fig. 3j). In order to investigate the functional role of the N-terminal domain, we performed a plasmid DNA silencing assay using the mutants K49A/R52A, H62A and R97A, and found that these mutations significantly reduced DNA silencing activity compared to wild-type *Rs*Ago (Fig. 5b). To confirm the importance of the N-terminal domain, we truncated an α-helix-β-stand segment on the edge of the N-terminal domain (P45–W63, Fig. 5a), which contacts nucleotides 15′ and 16′ of the target DNA strand, and performed a plasmid DNA silencing assay. This assay showed that the truncation mutation reduced plasmid DNA silencing activity to a greater extent than the point mutations (Fig. 5b).

On the basis of the results of the DNA silencing assay, we carried out an electrophoretic mobility shift assay using the *Rs*Ago-guide RNA (18-base) complex and ^32^P-radiolabelled target DNA strand (70-base, Supplementary Table 1) to analyze the binding activity of the truncation mutant ΔP45–W63 to target DNA. The ΔP45–W63 truncation mutant showed no binding to its target DNA (Fig. 5c), despite the mutant having the same binding capacity for 5′P guide strand RNA as wild-type *Rs*Ago (Supplementary Fig. 1a,l). These results indicate that the N-terminal domain is responsible for the binding of the target DNA strand to the *Rs*Ago–guide RNA complex.

### The heteroduplex segment required for recognition by *Rs*Ago

To identify the structural feature of the RNA/DNA heteroduplex that binds to *Rs*Ago, we analyzed the minor groove width using CURVES+^44^. In general, RNA/DNA duplexes adopt an A-form similar to double-stranded RNA^45^,^46^. However, the minor groove width of the RNA/DNA duplex structure bound to *Rs*Ago was mid-way between the standard values for the A- (11 Å) and B-forms (7.4 Å) (Supplementary Fig. 9a,b). A notable feature was that the width of the minor groove at nucleotides 12:12′ was clearly narrower than the standard value for the B-form. In general, sugar puckers in the A-form and B-form are predominately in C3′-endo and C2′-endo, respectively. In the RNA/DNA heteroduplex bound to *Rs*Ago, the guide RNA strand maintained a strong preference for C3′-endo sugar puckering, whereas the target DNA strand showed various sugar puckering conformations (C2′-endo, C1′-exo and O4′-endo), probably due to interaction with *Rs*Ago (Supplementary Table 2). Other geometrical parameters are shown in Supplementary Table 2. The most notable feature was that helical parameters of *Tip* and *Buckle* at nucleotides 12:12′ have large negative values. In addition, the *Roll* angle from the 11:11′ to 12:12′ step was large because of the bend of the duplex structure between nucleotides 11:11′ and 12:12′ (Supplementary Table 2 and Supplementary Fig. 9c). This characteristic bending may be promoted by the interaction of nucleotides beyond 12:12′ with the N-terminal and the PIWI domains.

**Figure 6:**
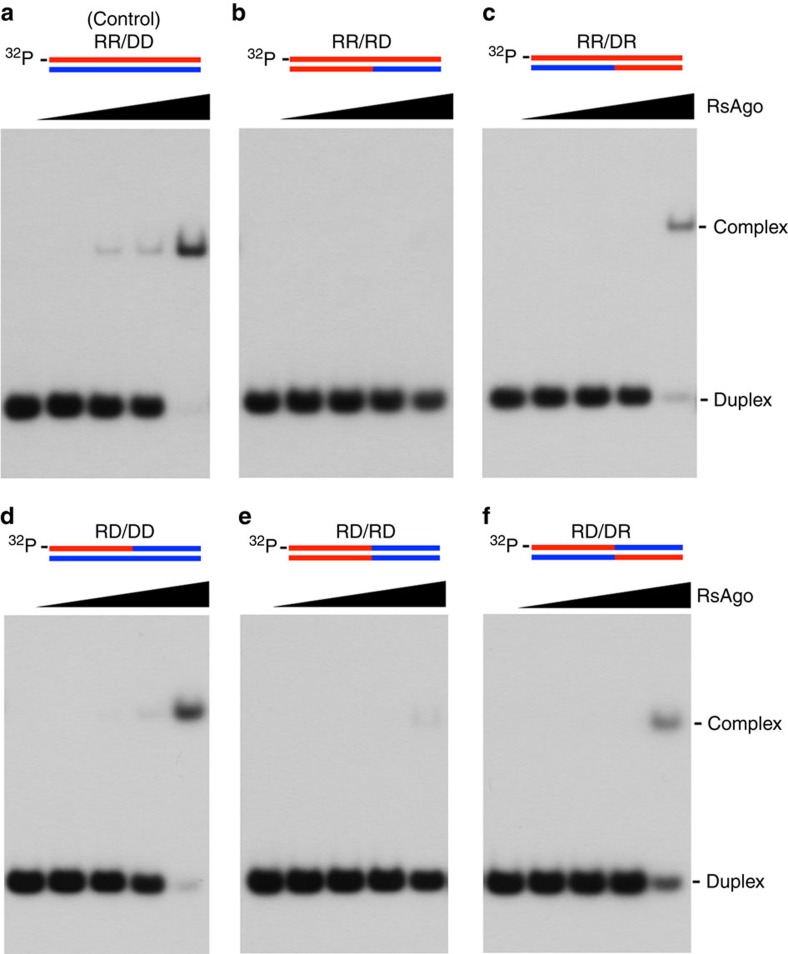
The guide RNA/target DNA heteroduplex region essential for binding to *Rs*Ago. (**a–f**) Schematic representation of the guide and target strands in the duplex is given at the top of each gel panel. RNA and DNA components are shown in red and blue, respectively. Binding affinities of the 18-base chimeric duplex to *Rs*Ago were examined using an electrophoresis mobility shift assay. The oligonucleotide sequences of the chimeric hybrid duplexes are shown in Supplementary Table 3. The positive control heteroduplex RR/DD (**a**) that normally binds to *Rs*Ago protein was incubated with various concentrations (0, 1, 10, 100 and 1,000 nM) of recombinant *Rs*Ago protein. Chimeric hybrid duplexes of RR/RD (**b**), RR/DR (**c**), RD/DD (**d**), RD/RD (**e**) and RD/DR (**f**) were incubated in the same way as the positive control RR/DD heteroduplex.

Previous studies showed that various Argonaute proteins bind to distinct types of nucleic acid, such as guide RNA/target RNA (all eukaryotes) and guide DNA/target DNA (most prokaryotes); here, we have identified a guide RNA/target DNA in *R. sphaeroides* (Figs 1 and 2). However, the selection mechanisms for guide/target strand types by each Argonaute protein are unknown. To determine which part of the guide/target strands is responsible for recognition by *Rs*Ago, we performed an electrophoresis mobility shift assay using chimeric nucleic acids with a boundary between positions 11:11′ and 12:12′ based on the unique helical conformation of the heteroduplex bound to *Rs*Ago, that is, the chimeric duplexes RR/RD and RR/DR, in which parts of 1′–11′ and 12′–18′ of the target DNA were replaced with RNA, respectively (Supplementary Table 3). Duplex recognition by *Rs*Ago was disturbed by the replacement of the 1′–11′ region of the target DNA with RNA (RR/RD), whereas recognition was maintained following replacement of the 12′–18′ region with RNA (RR/DR) (Fig. 6a–c). These results suggest that the 1′–11′ region of the target DNA is important for selection of strand type. To confirm this conclusion, we tested *Rs*Ago binding to the other chimeric duplexes (Fig. 6d–f and Supplementary Table 3). Although the full-length guide DNA/target DNA duplex showed no binding to *Rs*Ago (Fig. 1f), the RD/DD duplex, but not the RD/RD duplex, could bind to *Rs*Ago (Fig. 6d,e). Moreover, the RD/DR duplex retained the *Rs*Ago-binding ability (Fig. 6f). Overall, it is likely that a heteroduplex structure composed of the 1–11 region of guide RNA and the 1′–11′ region of target DNA is required for recognition by *Rs*Ago.

## Discussion

The binding assays performed in this study showed that *Rs*Ago specifically recognized RNA as a guide strand and DNA as a target strand (Figs 1 and 2). The structure of *Rs*Ago in complex with the heteroduplex provides some insight into the mechanism of this specific guide and target recognition. *Rs*Ago formed hydrogen bonds with the 2′-hydroxyl groups of 8 of the 18 nucleotides of the guide RNA strand (Fig. 2c). This binding to 2′-hydroxyl groups might contribute toward the ability of *Rs*Ago to specifically recognize RNA as a guide strand. Point mutations of almost all the amino acid residues involved in the recognition of 2′-hydroxyl groups did not show marked effects on plasmid DNA silencing activity (Figs 3c and 4a and Supplementary Fig. 7c). Therefore, we infer that the amino acid residues of *Rs*Ago, which recognize 2′-hydroxyl groups of the guide RNA, act cooperatively and contribute to the selection of the nucleic acid type of the guide strand.

**Figure 7:**
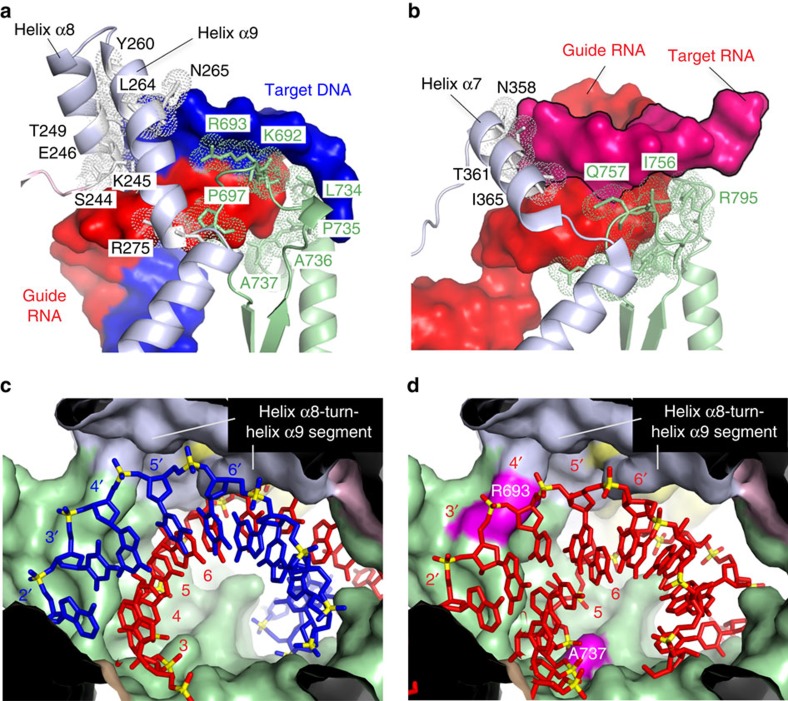
Interaction between the L2 linker and the PIWI domain of Argonaute and the seed region of the heteroduplex. (**a**) Recognition of the seed region in the guide RNA/target DNA heteroduplex by *Rs*Ago. The guide RNA (red) and target DNA (blue) strands are shown in surface representation. The L2 linker (light grey) and the PIWI domain (green) are shown in cartoon representation. Amino acid residues that interact with the heteroduplex are shown as sticks with surrounding dotted regions. Helices α8 (residues K245–L256) and α9 (N259–R275) are indicated with lines. (**b**) Recognition of the seed region in the guide RNA/target RNA duplex by *Hs*Ago2 (PDB ID: 4W5O). The guide RNA and partial target RNA strands are indicated in red. Helix α7 corresponding to helices α8 and α9 of *Rs*Ago is indicated with a line. (**c**,**d**) Steric hindrance between *Rs*Ago and the guide RNA/target RNA duplex in the seed region. (**c**) Structure of *Rs*Ago in complex with guide RNA/target DNA heteroduplex. *Rs*Ago is shown in surface representation and colour-coded as in Fig. 2a. The guide RNA and target DNA are shown as red and blue sticks, respectively, with phosphorous atoms in yellow. (**d**) Surface representation of the *Rs*Ago structure docked with the guide RNA/target RNA duplex (red) in the *Hs*Ago2 complex (PDB ID: 4W5O). The regions of *Rs*Ago that clash with the duplex are coloured in purple.

To investigate the mechanism of *Rs*Ago target strand recognition, we examined the interaction between *Rs*Ago and the minor groove of the seed region of the heteroduplex. Schirle *et al*.^15^ suggested that this interaction is crucial for target binding in *Hs*Ago2. The structure determined here showed that *Rs*Ago interacted with the minor groove of the seed region and with its periphery (Fig. 7a), as is the case in *Hs*Ago2 (Fig. 7b). Specifically, the helix α8-turn-helix α9 segment in the L2 linker of *Rs*Ago (S244, K245, E246, T249, Y260, L264, N265 and R275), which corresponds to helix α7 of *Hs*Ago2, and two loops in the PIWI domain (K692, R693, P697 and L734–A737), which correspond to the loop containing I756 and Q757 and the loop containing R795 in the PIWI domain of *Hs*Ago2, respectively, interacted with the minor groove and the backbone of the heteroduplex (nucleotides 4:4′–8:8′), making several hydrogen bonds and extensive van der Waals interactions (Fig. 7a,b and Supplementary Fig. 3b–d,h).

Considering the similarity of the recognition structures in the seed regions and also the conformational differences of the minor groove between the RNA/DNA duplex (Fig. 7a) and RNA/RNA duplex (Fig. 7b), we suggest that *Rs*Ago (and possibly other Argonaute proteins) might distinguish the nucleic acid type of the target strand through examination of the conformation of the minor groove of the seed region.

Next, we docked an RNA/RNA duplex, which bound to *Hs*Ago2, to *Rs*Ago. We focused on the interaction with the 1:1′–11:11′ region of the duplex because this region is crucial for recognition by *Rs*Ago (Fig. 6). The docking model showed that, unlike the case of target DNA (Fig. 7c), nucleotides 3′ and 4′ of the target RNA clashed with the area around R693, and that nucleotide 5 of the guide RNA clashed with the area around A737 in the PIWI domain (Fig. 7d). In addition, the helix α8-turn-helix α9 segment was separated from the seed region of the duplex (compare Fig. 7d with Fig. 7c). These shape mismatches for the guide RNA/target RNA duplex might also explain why *Rs*Ago specifically recognizes DNA as the target strand.

Two possible mechanisms can be suggested for the selection of 5′U-RNAs at the guide strand loading stage: first, the guide RNA is introduced to *Rs*Ago by an unknown loading machinery that selects 5′U-RNAs, and second, that *Rs*Ago itself selects the 5′U-RNAs from the pool of available sequences as the guide strand. Olovnikov *et al*.^37^ suggested the latter possibility was more likely as the heterologous expression of *Rs*Ago in *E. coli* cells results in the specific loading of 5′U-RNAs, as occurs for expression in *R. sphaeroides* cells. We concur with their conclusion because our structural and biochemical data showed that *Rs*Ago preferably interacted with 5′U-guide RNA (Fig. 3a,b and Table 1). Moreover, the *R. sphaeroides* gene shows no homologies to Dicer or TRBP/PACT sequences, that is, small RNA-processing proteins^47^. We therefore conclude that *Rs*Ago itself directly selects 5′U-RNAs from the available pool of sequences, without the aid of any loading machinery.

**Figure 8:**
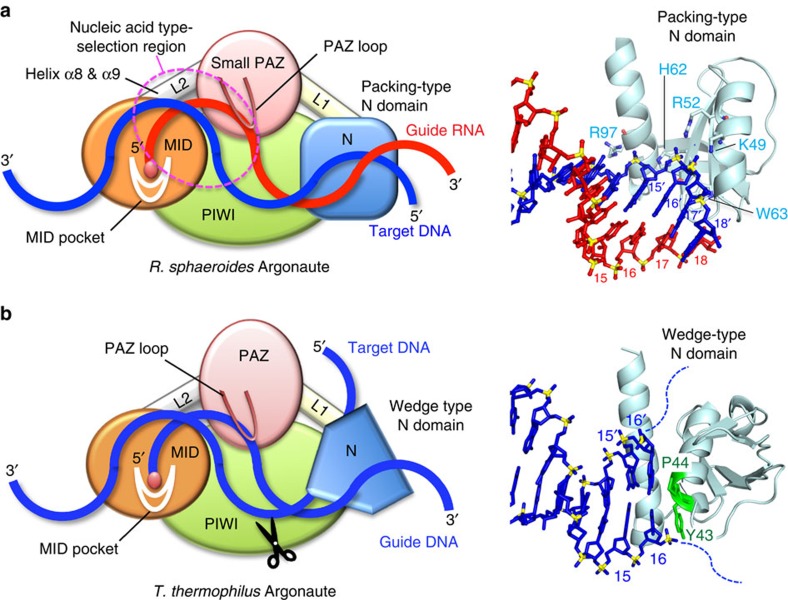
A model for target-binding mode of Argonaute. (**a**) The target recognition model of the ‘Packing type' provided by the N-terminal domain of *Rs*Ago (left panel). Nucleic acid type-selection region is shown in a dashed-red circle. The right panel shows a close-up view of the interaction between the 18-base guide RNA (red) and the 18-base target DNA (blue) heteroduplex and the N-terminal domain (light blue) of *Rs*Ago. Amino acid residues that interact with the target strand are shown in stick representation. The N-terminal domain of *Rs*Ago allows base-pair formation at the guide strand 3′-region. (**b**) The target recognition model of the ‘Wedge type' provided by the N-terminal domain of *Tt*Ago (left panel). The right panel shows a close-up view of the interaction between the 21-base guide DNA (blue), 19-base target DNA (blue) and the N-terminal domain (light blue) of *Tt*Ago (PDB ID: 4KXT). Nucleotide 16 of the guide strand and nucleotide 16′ of the target strand stack on Y43 and P44 (light green), respectively. The N-terminal domain of *Tt*Ago blocks the base-pairing propagation of the guide/target duplex beyond position 16:16′.

In the present study, we solved the structure of *Rs*Ago in a complex with a guide RNA/target DNA heteroduplex. Surprisingly, the structure showed that base pairing in the duplex is maintained in the 3′-region of the guide strand (nucleotides 14–18) by the packing provided by the N-terminal and the PIWI domains (Fig. 2). In addition, we obtained biochemical data, which suggested that this packing is crucial for target binding and silencing (Fig. 5b,c). On the basis of these data, we present a model for the target-binding mode of *Rs*Ago (Fig. 8a). In this model, the 3′-region of the guide strand (nucleotides 14–18) base pairs with the 5′-region of the target strand (nucleotides 14′–18′); this base pairing is stabilized by the cooperative action of the N-terminal and PIWI domains. This model is consistent with the fact that *Rs*Ago-associated RNAs are derived from cellular transcripts that lack distinct secondary structures. That is, in this situation, there is no need to release the passenger strand for target binding; therefore, the N-terminal domain of *Rs*Ago does not have to function as a wedge to unwind the duplex, unlike *Tt*Ago and *Hs*Ago2 (Fig. 8b)^9^,^29^. Furthermore, our model can clearly explain the recently described behaviour in which the *Rs*Ago–RNA complex loaded onto a complementary foreign target DNA (such as transposons, phage genes or exogenous plasmids) could inhibit RNA polymerase loading or RNA polymerase elongation, leading to the repression of transcription of the target DNA^37^. In this situation, it seems likely that, in addition to the base pairing in the seed region, base pairing at the 3′-region of the guide strand (nucleotides 14–18) might contribute to the strong interaction with the target strand. In conclusion, our present study of *Rs*Ago represents a significant advance toward the complete understanding of the molecular mechanism of RNA-guided DNA silencing.

## Methods

### Plasmid constructions

We constructed expression plasmids encoding full-length *Rs*Ago (residues 1–777). DNA fragments encoding the full-length Argonaute gene were amplified by PCR from the genomic DNA of *R. sphaeroides* (ATCC No. 17025D-5) using KOD plus NEO DNA polymerase (TOYOBO). The PCR product was cloned between the NdeI and XhoI sites of the pET-28a (Novagen) and thus the construct contained the sequence MGSSHHHHHHSSGLVPAGSH (6 × His-tag and thrombin-recognition sequence) in the N terminus. Point and deletion mutants were generated in *Rs*Ago plasmids by PCR site-directed mutagenesis^48^ using a pET-28a plasmid containing the *Rs*Ago gene as the template (Supplementary Table 4). These modified Argonaute gene sequences in pET-28a were verified by DNA sequencing.

### Protein expression

*Rs*Ago wild-type (WT) and mutants were overexpressed in *E. coli* BL21 (DE3) cells (Novagen). The transformed *E. coli* cells were grown in LB medium containing 15 mg l^−1^ kanamycin to an OD_600_ of 0.6 at 37 °C and expression was induced with 0.5 mM isopropyl *β*-D-1-thiogalactopyranoside. The cells were further cultured at 25 °C for 18 h and collected by centrifugation. To perform the crystal structure analysis, a Selenomethionine (SeMet)-labelled *Rs*Ago NΔ20 mutant was overexpressed in *E. coli* B834(DE3) cells in SeMet core medium (Wako) supplemented with 20 g l^−1^ D-glucose, 1 × MEM Vitamin solution (Sigma), 250 mg l^−1^ MgSO_4_, 4.2 mg l^−1^ FeSO_4_, 15 mg l^−1^ kanamycin and 50 mg l^−1^ L-SeMet (Wako).

### Protein purification

The *E. coli* cells expressing WT and mutant *Rs*Ago proteins were resuspended in ice-cold buffer A (50 mM HEPES-KOH pH 7.5, 1 M ammonium chloride, 5% glycerol and 5 mM β-mercaptoethanol) and disrupted by sonication. The lysate was clarified by centrifugation at 100,000 g for 30 min. The protein was first purified using Ni-NTA agarose (Qiagen) and eluted with buffer A containing 300 mM imidazole. The eluted protein was dialyzed against buffer B (20 mM Tris–HCl, pH 7.6, 300 mM NaCl and 5 mM *β*-mercaptoethanol) and passed through a Resource Q column (GE Healthcare). The protein was concentrated using an Amicon Ultra 10 K filter (Millipore) and purified by chromatography on a HiLoad 26/60 Superdex 200 pg column (GE Healthcare). The purified protein was dialyzed against buffer C (20 mM Tris–HCl, pH 7.6, 500 mM NaCl and 5 mM *β*-mercaptoethanol) and concentrated using an Amicon Ultra 10 K filter. The protein samples were stored at −80 °C. SeMet-labelled *Rs*Ago ΔN20 was purified in the presence of 10 mM DTT using a protocol similar to that used for WT *Rs*Ago.

### Preparation of oligonucleotides

The crystallization and biochemical analysis were carried out using synthetic oligonucleotides. They were purchased from GeneDesign, Inc. (Osaka, Japan). The sequences are shown in Supplementary Tables 1 and 3.

### Crystallization

For crystallization, *Rs*Ago:guide RNA:target DNA complexes were formed by mixing *Rs*Ago (ΔN20) (native or SeMet-labelled), 18-base 5-phosphorylated RNA and 18-base DNA in a 1:1.2:1.2 molar ratio in a buffer containing 10 mM Tris–HCl, pH 7.6, 200 mM NaCl and 5 mM MgCl_2_ at 20 °C for 30 min. Crystallization drops were prepared by mixing 1 μl of the complex solution (10 mg ml^−1^ of *Rs*Ago) and 1 μl of a reservoir solution containing 0.05 M sodium cacodylate trihydrolate (pH 7.0 for native *Rs*Ago and pH 7.4 for SeMet-labelled *Rs*Ago) and 2-methyl-2,4-pentanediol (42% (v/v) for native *Rs*Ago and 45% (v/v) for SeMet-labelled *Rs*Ago). These crystals were grown at 20 °C using sitting-drop vapor-diffusion methods in a 24-well VDX plate (Hampton Research).

### Diffraction data collection

For data collection, crystals were cryoprotected by a reservoir solution containing 25% (v/v) glycerol and then flash-cooled at 100 K. Datasets of native and SeMet derivatives (at the anomalous peak wavelength) were collected at beamline NW12A of KEK PF-AR (Tsukuba, Japan) using an ADSC Quantum 210r CCD detector, and were processed with the program HKL2000^49^ and the CCP4 program suite^50^.

### Structure determination

The structure was determined by the single-wavelength anomalous dispersion method. The locations of Se sites, refinement and phasing calculations were performed using the program autoSAHRP^51^. Twenty-eight Se sites were identified in the asymmetric unit, and the Figure of Merit for acentric and centric was 0.33151 and 0.12404, respectively. Subsequently, density modification was performed using the program SOLOMON^52^. The initial model was built with the program BUCCANEER^53^, followed by model improvement against the native dataset using the program ARP/wARP^54^. The model was further improved by iterative cycles of manual model building with the program COOT^55^ and maximum likelihood refinement with the program REFMAC5^56^. In the Ramachandran plot, 95.9% of the residues were included in the favored region, 3.4% were in the allowed region and 0.8% were in the outlier region. The statistics for data collection and refinement are summarized in Table 2. A representative 2*F*_o_−*F*_c_ electron density map is displayed in Supplementary Fig. 10.

### Electrophoretic mobility shift assay

*Rs*Ago and ^32^P-labelled nucleic acids were incubated in 10 μl of binding buffer containing 20 mM HEPES-KOH, pH 7.5, 200 mM NaCl and 5 mM MgCl_2_ for 10 min at 25 °C. The concentration of radiolabelled nucleotides was fixed as 5 nM, whereas the concentration of *Rs*Ago varied. After incubation, the reaction samples were mixed with 1 μl dye solution containing 50% glycerol, 0.1% bromophenol blue and 0.1% xylene cyanol. The nucleoprotein complexes were fractionated by electrophoresis (100 V, 1 h) through 6% native polyacrylamide gels (39:1 acrylamide/bisacrylamide) with 5 mM MgCl_2_ in Tris–glycine buffer (25 mM Tris, 192 mM glycine) and were detected by autoradiography. Full-size images of the most important blot are presented in Supplementary Fig. 11.

### Plasmid DNA silencing assay with *E. coli* cells

*E. coli* BL21(DE3) cells containing expression plasmids (pET-28a) of *Rs*Ago WT and mutants were grown in LB medium at 37 °C with kanamycin (15 μg ml^−1^) to the log phase (OD_600_=0.5). After *β*-D-1-thiogalactopyranoside (0.5 mM) induction, the cells were further incubated for 5 h at 30 °C. The expression levels of all *Rs*Ago variants were checked by SDS–PAGE. After the cell culture, plasmids were isolated using QIAprep Spin Miniprep Kit (Qiagen). The concentration of plasmid DNA was determined with a NanoDrop spectrophotometer (Thermo Scientific). This experiment was based on Olovnikov *et al*.^37^

### Fluorescence polarization assay

Fluorescence polarization assays^57^ to measure the interactions of *Rs*Ago and nucleic acids were performed using a Pan Vera Beacon 2000 fluorescence polarization instrument (Invitrogen). Polarization values were measured using a buffer containing 20 mM HEPES-KOH, pH 7.5, 200 mM NaCl, 5 mM MgCl_2_, 0.01 mg ml^−1^ *E. coli* total-tRNA and 0.01% NP-40. To obtain a titration curve, increasing amounts of *Rs*Ago were added to the buffer with 10 pM of 3′-6-FAM-labelled nucleotide. The mixed samples were incubated for 10 min at 25 °C before measurement and the polarization value was measured successively at 25 °C. The data were fitted to a nonlinear regression using the one site specific binding function with GraphPad Software Prism 6.

### Data availability

The crystal structure of *Rs*Ago in complex with guide RNA and target DNA has been deposited in the RCSB Protein Data Bank (PDB) under PDB ID code 5AWH. All other data that support the findings of this study are available from the corresponding author upon reasonable request.

## Additional information

**How to cite this article**: Miyoshi, T. *et al*. Structural basis for the recognition of guide RNA and target DNA heteroduplex by Argonaute. *Nat. Commun.* 7:11846 doi: 10.1038/ncomms11846 (2016).

## Supplementary Material

Supplementary Information Supplementary Figures 1-11, Supplementary Tables 1-4 and Supplementary Notes.

## Figures and Tables

**Table 1:** Equilibrium dissociation constant (Kd) values of *Rs*Ago for 18-base single strand RNA and DNA

RsAgo	Nucleic acid	Kd (nM)
WT	5′P-RNA (5′U-RNA)	0.91±0.06
WT	5′P-DNA	42.6±3.4
WT	5′OH-RNA	1,144±54
WT	5′P-RNA (with EDTA)	7.71±0.36
WT	5′A-RNA	24.8±1.7
WT	5′C-RNA	67.0±3.5
WT	5′G-RNA	181.3±13.3
Y463A/K467A	5′P-RNA	1,697±123
R481A/T484A	5′P-RNA	718±36
K506A	5′P-RNA	1,034±47
ΔL777	5′P-RNA	38.7±2.1

The Kd values were measures by a fluorescence polarization assay using nucleic acids modified with 6-carboxyfluorescein (6-FAM) at the 3′-end position. The nucleic acid sequences are shown in Supplementary Table 1.

**Table 2:** Data collection, phasing and refinement statistics

	Native	SeMet
**Data collection**		
Space group	*P*2_1_	*P*2_1_
Cell dimensions		
*a*, *b*, *c* (Å)	68.2, 118.3, 118.5	67.6, 116.7, 117.7
*α*, *β*, *γ* (°)	90.0, 95.8, 90.0	90.0, 95.6, 90.0
Wavelength	1.0	0.97921
Resolution (Å)	50–2.00 (2.03–2.00)*	50–2.10 (2.14–2.10)
*R*_merge_† (%)	6.3 (40.0)	7.4 (43.7)
*I /* σ*I*	35.8 (3.7)	50.5 (6.0)
Completeness (%)	97.7 (100)	99.1 (100)
Redundancy	3.8 (3.8)	7.5 (7.3)
		
**Refinement**		
Resolution (Å)	35.6–2.00	
No. reflections	117,604	
*R*_work_/*R*_free_‡ (%)	18.4/23.3	
No. atoms		
Protein	11,669	
RNA/DNA/Mg^2+^	748/750/2	
Water	646	
*B*-factors		
Protein	59.5	
RNA/DNA/Mg^2+^	50.1/55.2/40.1	
Water	55.2	
R.m.s deviations		
Bond lengths (Å)	0.016	
Bond angles (°)	1.758	

^*^Values in parentheses are for the highest resolution shell.

^†^*R*_merge_= Σ_*hkl*_Σ_*i*_ |*I*_*i*_(*hkl*)−<*I*(*hkl*)>|/Σ_*hkl*_Σ_*i*_ *I*_*i*_(*hkl*), where *I*_*i*_(*hkl*) is the *i*-th intensity measurement of reflection *hkl*, including symmetry-related reflections, and <*I*(*hkl*)> is its average.

^‡^*R*_free_ was calculated by using 5% of randomly selected reflections that were excluded from the refinement.
