# Supplementary material for: Structural basis for the recognition of guide RNA and target DNA heteroduplex by Argonaute
Source: Nat Commun. 2016 Jun 21;7:11846. doi: 10.1038/ncomms11846 (PMC4919518; doi:10.1038/ncomms11846)
Supplement: Supplementary Information — Supplementary Figures 1-11, Supplementary Tables 1-4 and Supplementary Notes. [file ncomms11846-s1.pdf]

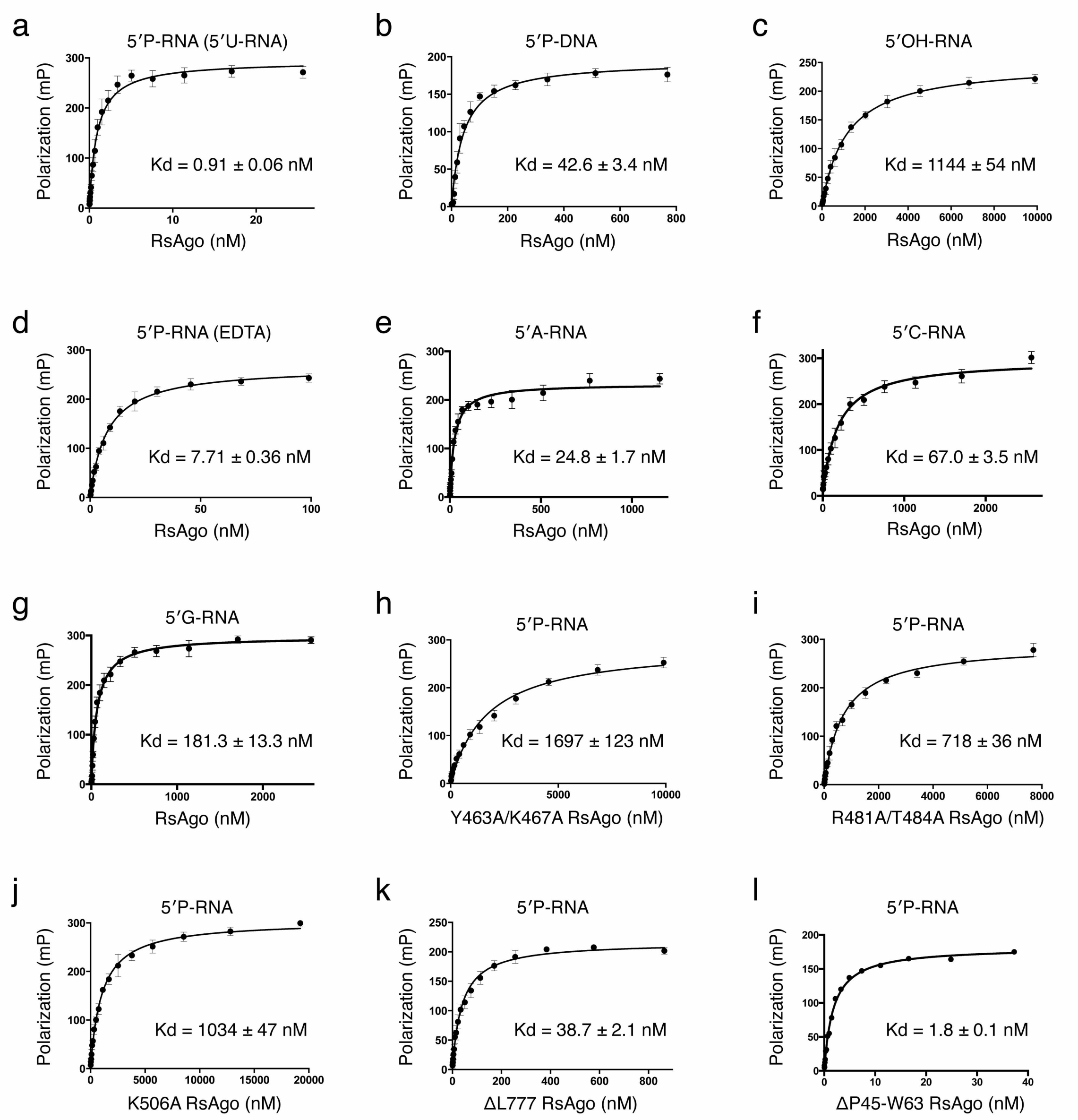

Supplementary Figure 1: Nucleic acid binding activities of *Rs*Ago protein measured by a fluorescence polarization assay. (**a-g**) Titration binding curves for wild-type recombinant *Rs*Ago protein with various 18-base nucleic acid ligands labeled with 6-carboxyfluorescein (6-FAM) at their 3′-ends were obtained by increasing the concentration of protein: (**a**) 5′-phosphorylated-RNA [5′P-RNA]; (**b**) 5′-phosphorylated DNA [5′P-DNA]; (**c**) 5′-hydroxylated RNA [5′OH-RNA]; (**d**) Binding activity of 5′-phosphorylated RNA was measured with buffer containing 5 mM EDTA instead of magnesium; (**e**) 5′A-phosphorylated RNA [5′A-RNA]; (**f**) 5′C-phosphorylated RNA [5′C-RNA]; (**g**) 5′G-phosphorylated RNA [5′G-RNA]. (**h-l**) Titration binding curve for recombinant Y463A/K467A (**h**), R481A/T484A (**i**), K506A (**j**), ΔL777 (**k**) and ΔP45-W63 (**l**) *Rs*Ago variants with 5′-phosphorylated 18-base RNA labeled with 6-FAM at the 3′-end was obtained by increasing the concentration of protein. The oligonucleotide sequences are shown in Supplementary Table 1. Error bars represent SD values (n = 3).

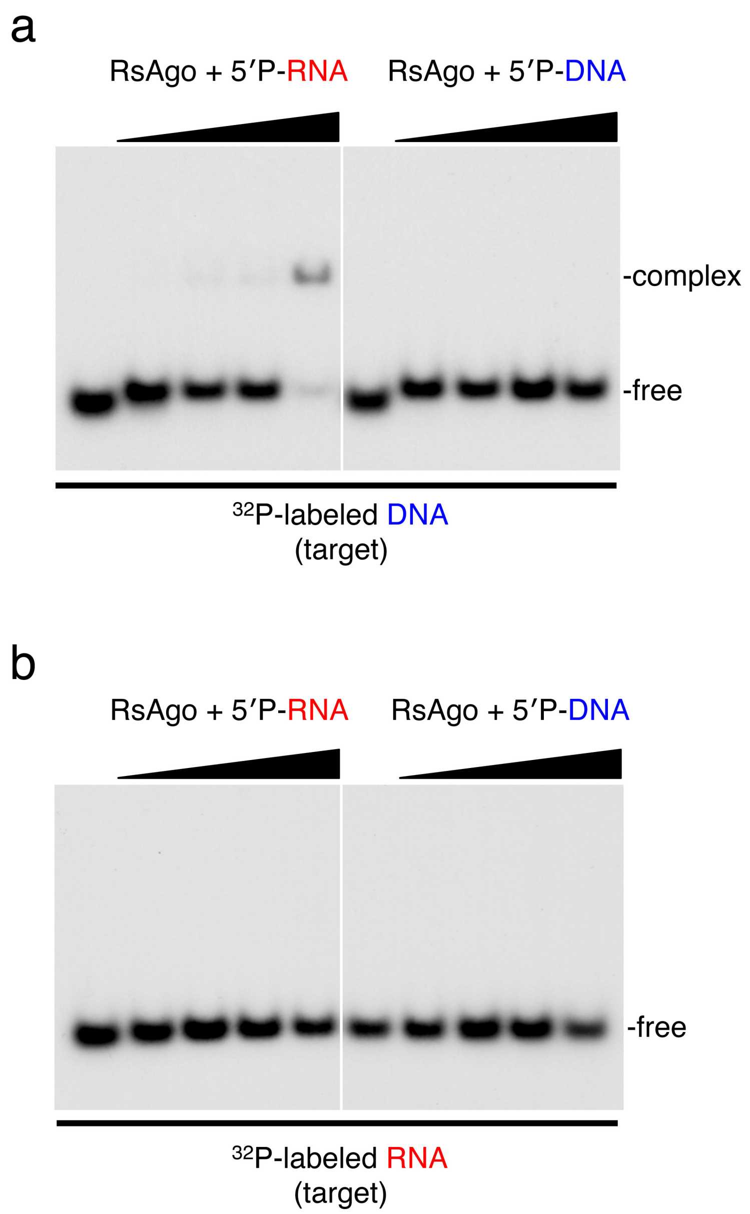

Supplementary Figure 2: Sequential binding assay to analyze binding of guide and target strands to *Rs*Ago. Electrophoresis mobility shift assay using the *Rs*Ago complex containing the guide (RNA or DNA) strands and the target DNA (**a**) or RNA (**b**) strands. Various concentrations (0, 1, 10, 100 and 1000 nM) of *Rs*Ago were incubated with the same concentrations of 5′-phosphorylated 18-base guide RNA (left panel) or DNA (right panel) strands, and then the mixed samples were incubated with 5 nM of ^{32}P-labeled 18-base target DNA (**a**) or RNA (**b**) strands.

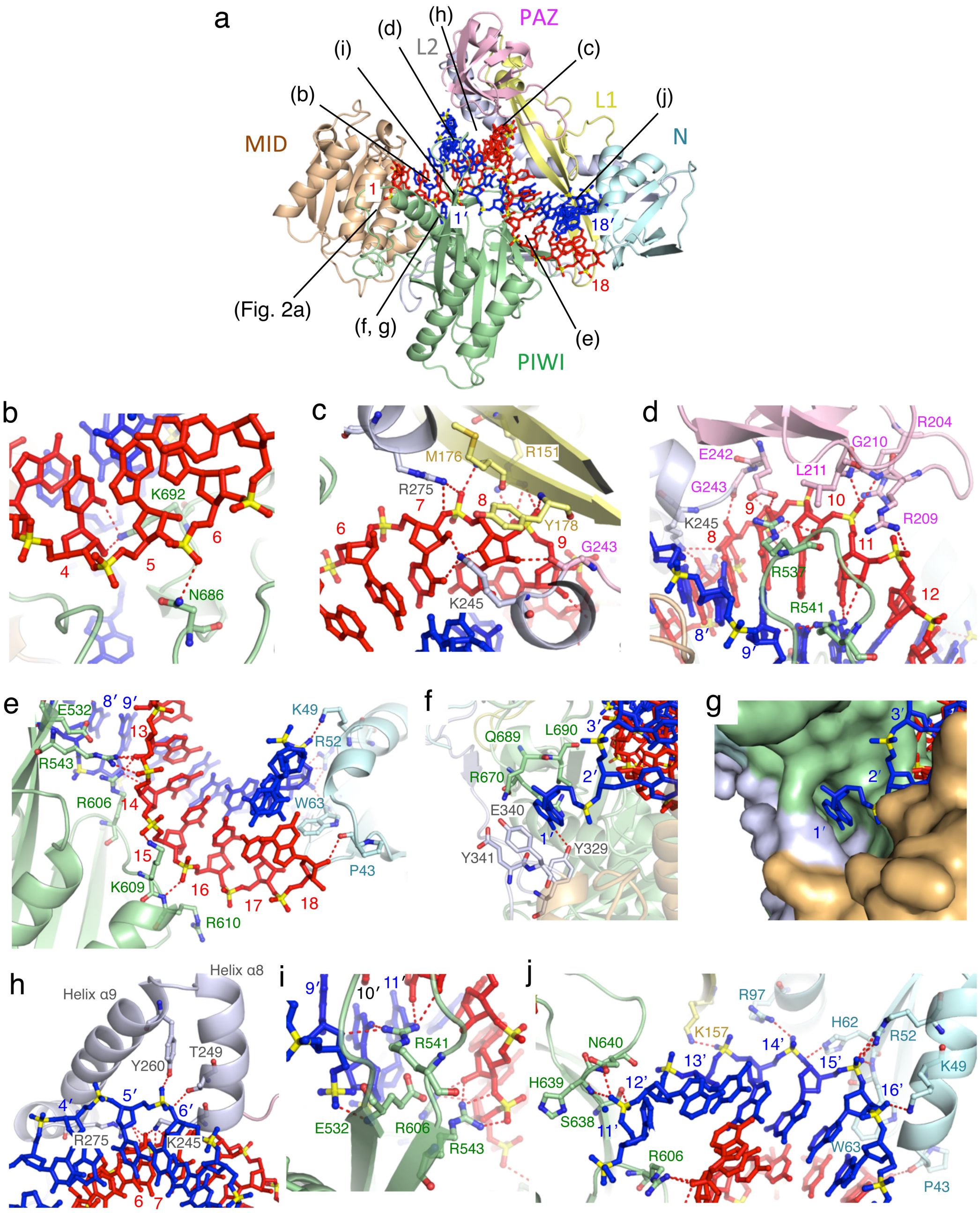

Supplementary Figure 3: Interactions between the *R. sphaeroides* Argonaute protein and an 18-base guide RNA/target DNA heteroduplex based on the crystal structure. (**a**) Overview of the crystal structure of *Rs*Ago bound to a 5′-phosphorylated 18-base RNA guide strand and 18-base DNA target strand. The guide RNA (red) and target DNA (blue) are shown in stick representation, with phosphorous atoms in yellow. Individual domains and linkers of the Argonaute protein are shown in cartoon representation, and with the same color-coding as Figure 2a. (**b-j**) Close-up view of the interaction between *Rs*Ago and the heteroduplex. Hydrogen bonds are shown as dashed red lines.

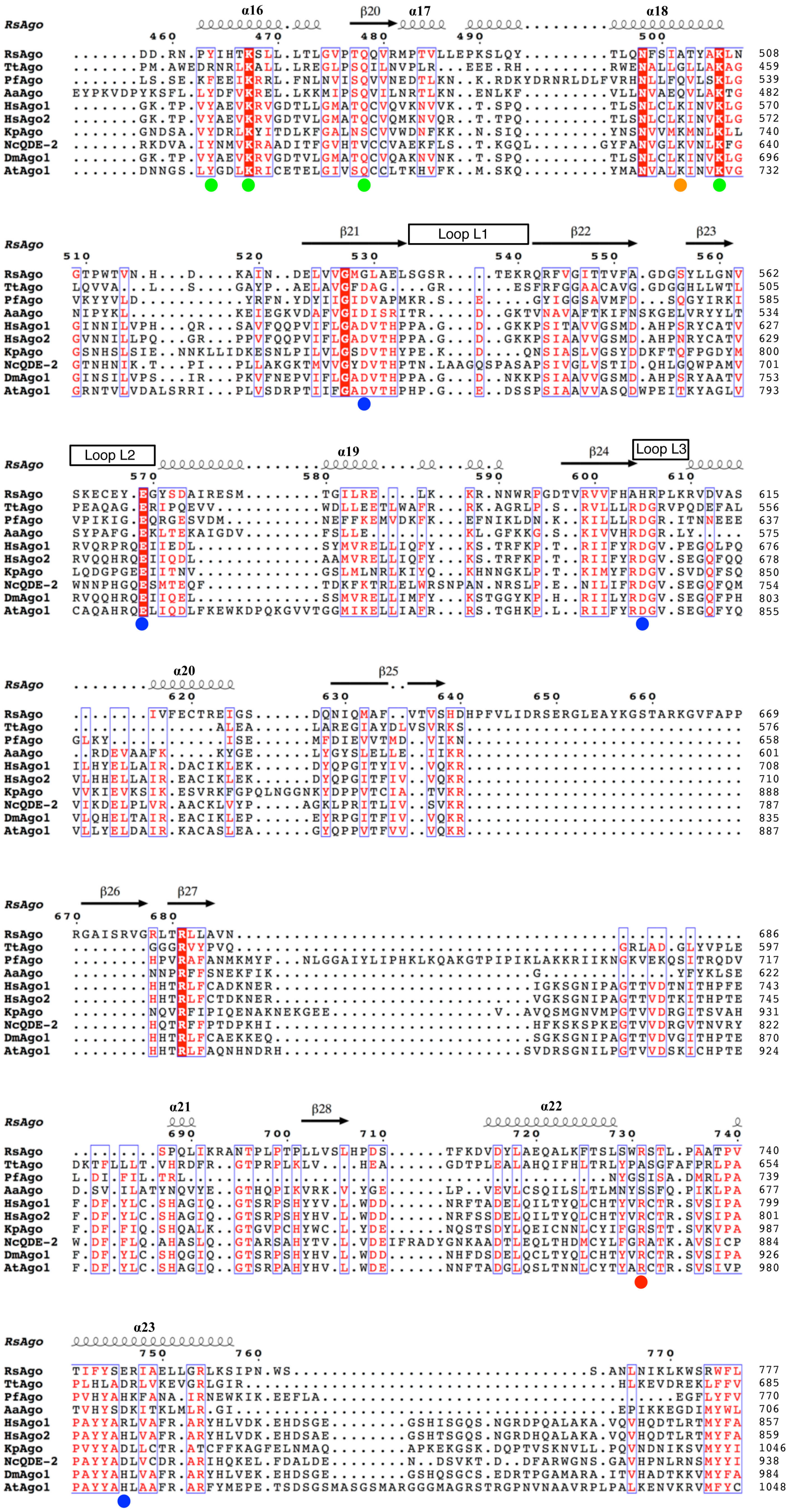

Supplementary Figure 4: The sequence alignment of Argonaute proteins. Protein sequences were aligned across their MID (partially) and PIWI domains. The aligned sequences

are in the order *Rhodobacter sphaeroides* Ago (*Rs*Ago), *Thermus thermophilus* Ago (*Tt*Ago), *Pyrococcus furiosus* Ago (*Pf*Ago), *Aquifex aeolicus* Ago (*Aa*Ago), human Argonaute2 (*Hs*Ago2), human Argonaute1 (*Hs*Ago1), *Kluyveromyces polysporus* (*Kp*Ago), *Neurospora crassa* QDE-2 Argonaute (*Nc*QDE-2), *Drosophila melanogaster* Argonaute1 (*Dm*Ago1) and *Arabidopsis thaliana* Argonaute1 (*At*Ago1). Residue numbers and the secondary structure of *Rs*Ago are indicated above the alignment. Conserved residues are shown in red, and essentially invariant residues are shaded in red. Conserved binding residues in the MID domain and slicer catalytic residues are shown by green and blue circles, respectively. The amino acid residue highly conserved in eukaryotic Argonautes corresponding to K566 in *Hs*Ago2 is shown by the orange circle. The amino acid residue that is highly conserved in RNA-guided Argonautes corresponding to R731 in *Rs*Ago is shown by the red circle.

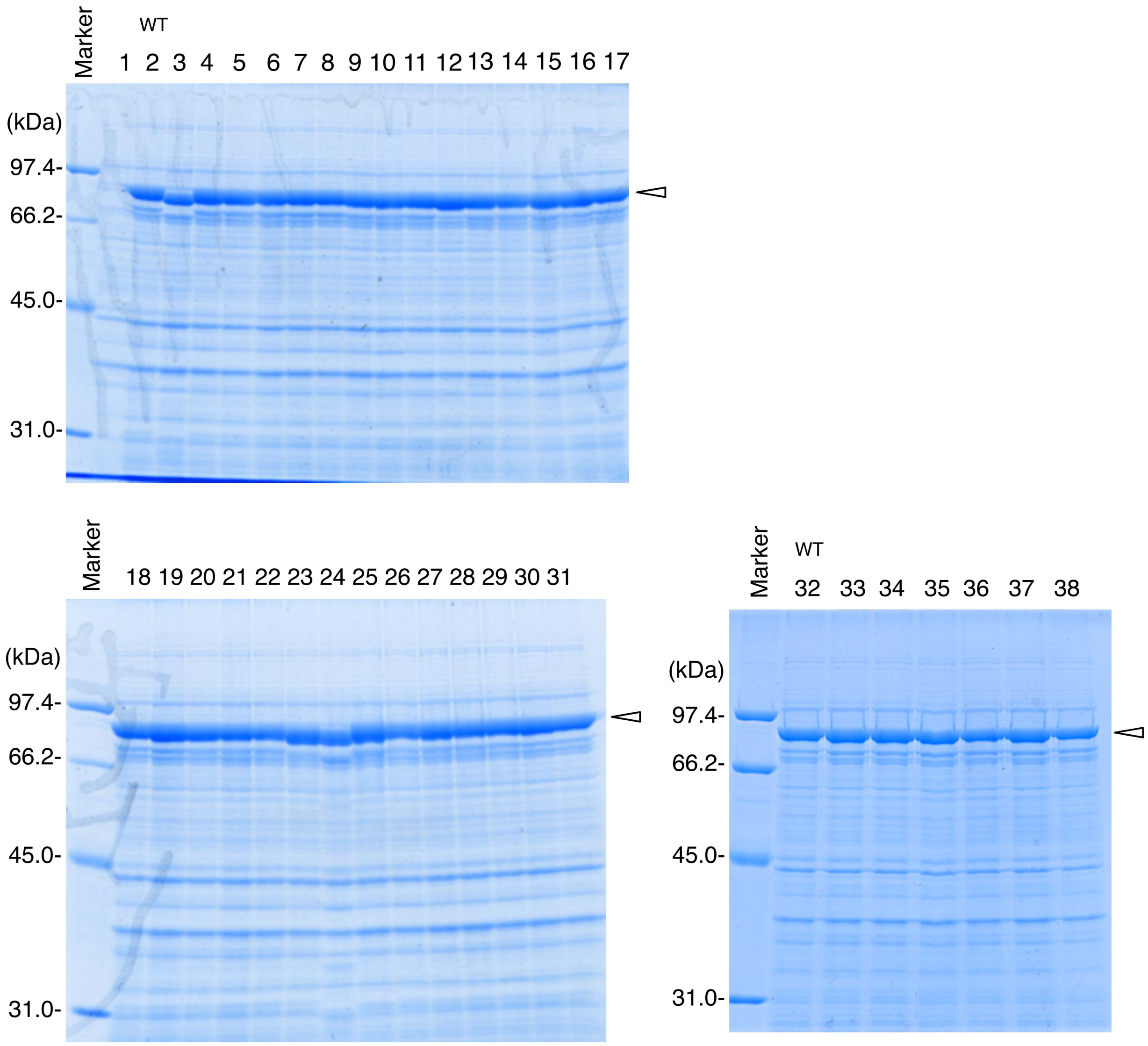

Supplementary Figure 5: Overexpression of *Rs*Ago WT and mutant derivative proteins. Whole cell lysates from native *E. coli* cells (lane 1) and His-tagged WT (lane 2), ΔN20 (lane 3), Y329A (lane 4), R754A (lane 5), ΔL777 (lane 6), Y463A/K467A (lane 7), R481A/T484A (lane 8), R204A/R209A (lane 9), S638A/H639A/D640A (lane 10), R151A (lane 11), E242A (lane 12), R275A (lane 13), Q478A (lane 14), K506A (lane 15), R606A (lane 16), N686A/K692A (lane 17), R731A (lane 18), R537A (lane 19), R541A (lane 20), R543A (lane 21), R537A/R541A/R543A (lane 22), ΔS536-R541 (lane 23), ΔP45-W63 (lane 24), K49A/R52A (lane 25), H62A (lane 26), R97A (lane 27), K157A (lane 28), K245A (lane 29), T249A (lane 30), Y260A (lane 31), WT (lane 32), R204A (lane 33), R209A (lane 34), Y463A (lane 35), K467A (lane 36), R481A (lane 37) and T484A (lane 38) *Rs*Ago protein expressing cells were run on 10% SDS-PAGE to confirm the level of *Rs*Ago expression. The expressed proteins are indicated by arrowhead.

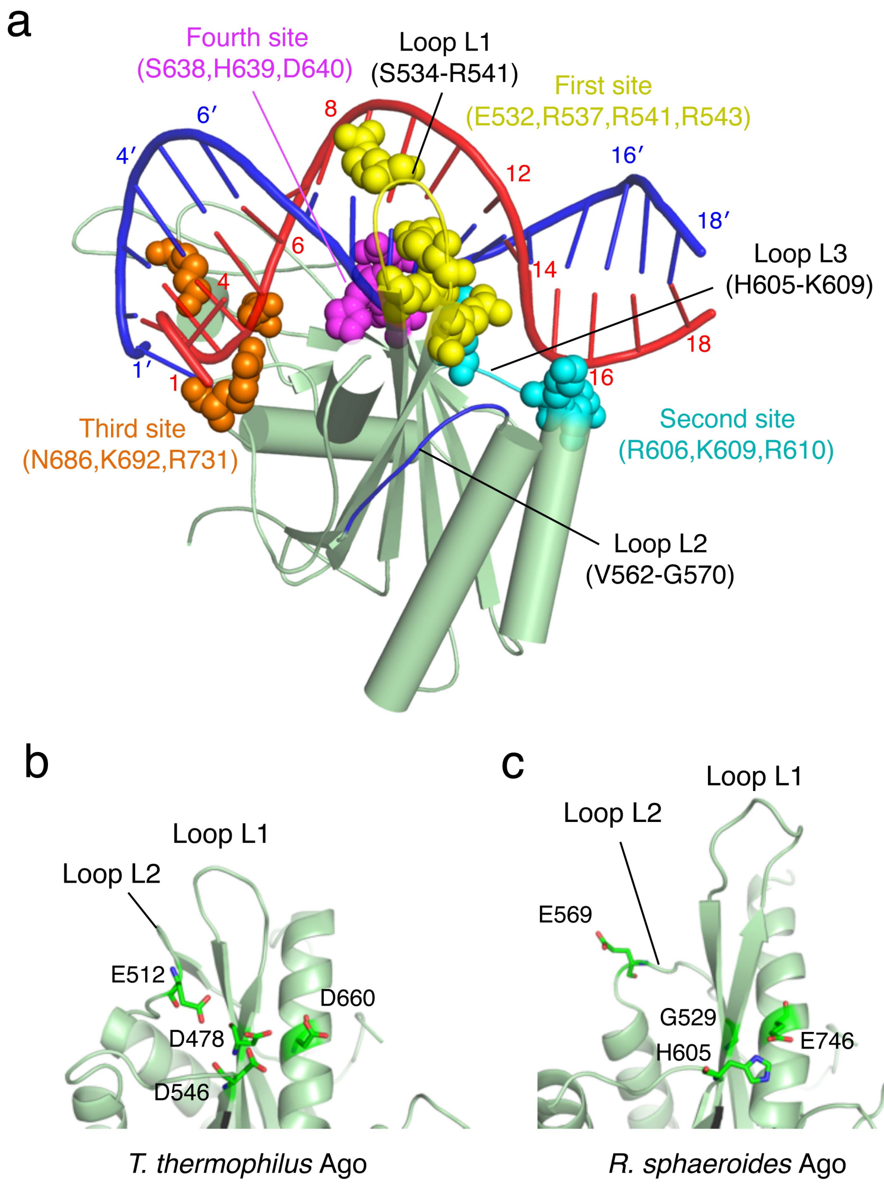

Supplementary Figure 6: Widespread interaction between the PIWI domain and the heteroduplex. (**a**) Close-up view of the PIWI domain (green) and guide RNA (red)/target DNA (blue) duplex in the *Rs*Ago complex. The first, second, third and fourth binding sites of the PIWI domain that interact with the heteroduplex are shown in yellow, cyan, orange and pink, respectively. Amino acid residues that directly interact with the heteroduplex are shown as spheres. The loops L1 (S534-R541), L2 (V562-G570) and L3 (H605-K609) are shown in yellow, blue and cyan, respectively. (**b**,**c**) Comparison of catalytic residues of *Rs*Ago and *Tt*Ago. (**b**) The catalytic center of *Tt*Ago (PDB ID: 4NCB). The four catalytic residues (D478, D546, D660 and E512) are shown in stick representation. (**c**) The equivalent region of *Rs*Ago corresponding to the catalytic center of *Tt*Ago. The four residues corresponding to the catalytic residues of *Tt*Ago (G529, H605, E746 and E569) are shown in stick representation.

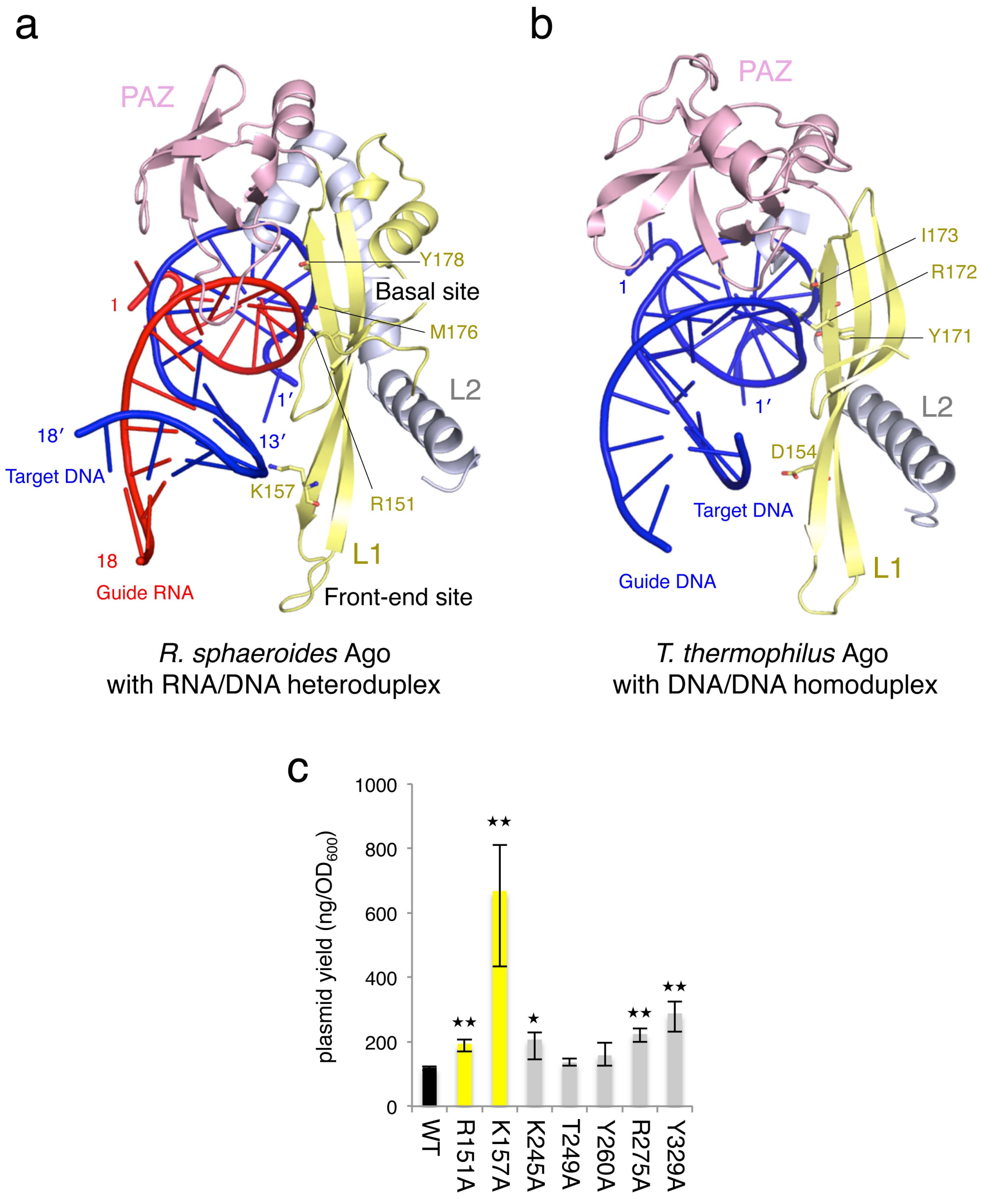

Supplementary Figure 7: Functional interactions between the L1/L2 linkers and the heteroduplex. (**a**) Close-up view of the L1 linker (yellow) and guide RNA (red)/target DNA (blue) duplex in *Rs*Ago complex. Amino acid residues of the L1 linker that interact with the heteroduplex are shown in yellow stick representation. (**b**) Close-up view of the L1 linker (yellow) and guide DNA/target DNA duplex (blue) in *Tt*Ago complex (PDB ID: 4NCB). Amino acid residues of the L1 linker that interact with the duplex are indicated with lines. The negatively charged amino acid D154 is also shown in yellow stick representation. (**c**) Effect of *Rs*Ago mutations in the L1 and L2 linkers on plasmid DNA silencing. Plasmid DNAs, which express various *Rs*Ago variants in *E. coli* cells, were purified and the plasmid yields were quantified. Error bars represent SD values (n = 3). *$P < 0.05$ and **$P < 0.01$ compared with WT using Student's *t*-test.

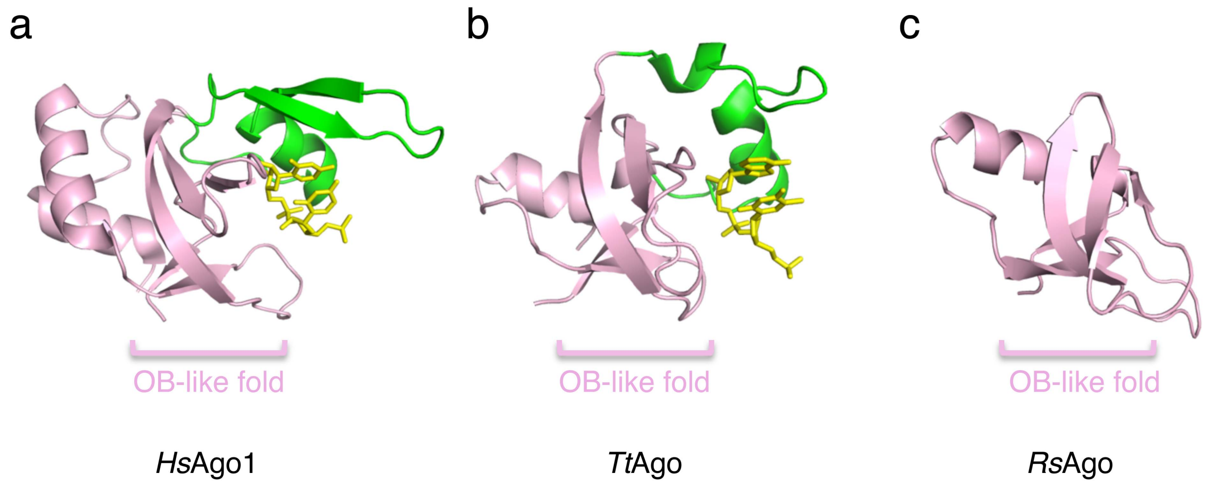

Supplementary Figure 8: Structural comparison of the PAZ domain from human, *T. thermophilus* and *R. sphaeroides* Argonautes. The PAZ domains of *Hs*Ago1 (**a**, PDB ID: 4KXT) *Tt*Ago (**b**, PDB ID: 4NCB) and *Rs*Ago (**c**) are shown in cartoon representation. One subdomain containing an OB-like fold is shown in pink, and a second subdomain is shown in green. The guide strand 3′-end nucleotides that bind to the PAZ pocket are shown in yellow stick representation.

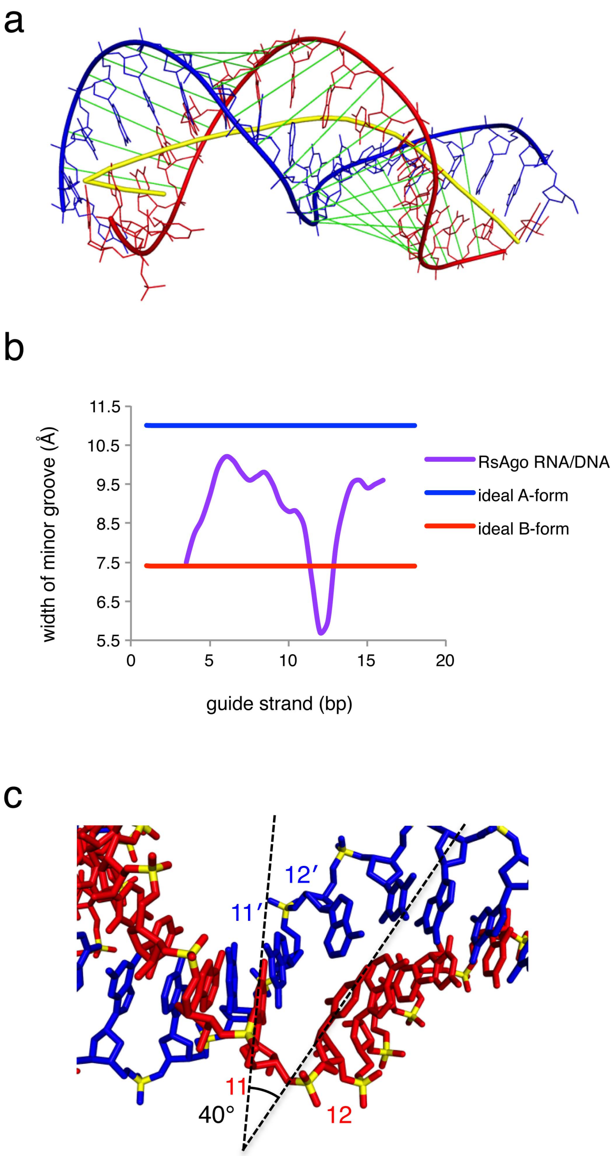

Supplementary Figure 9: Characteristic conformation of the 5′-phosphorylated 18-base RNA guide strand and 18-base DNA target strand bound to *R. sphaeroides* Argonaute. (**a**) Graphical output of a Curves+ analysis for RNA/DNA heteroduplex bound to *Rs*Ago. The RNA (red) and DNA (blue) strands are shown in line representations with the minor groove width vectors (green) and the calculated helical axis (yellow). (**b**) A plot of minor groove width for RNA/DNA heteroduplex bound to *Rs*Ago is shown in purple. The minor groove width of the ideal A-form and ideal B-form is shown in blue and red, respectively. The groove parameters were calculated using the CURVES+ program. (**c**) The helical structure of the middle region of 18-base heteroduplex bound to *Rs*Ago. The RNA (red) and DNA (blue) are shown in stick representations with phosphorous atoms in yellow. The bending angle of the heteroduplex structure is shown between positions 11 and 12 of the guide RNA strand.

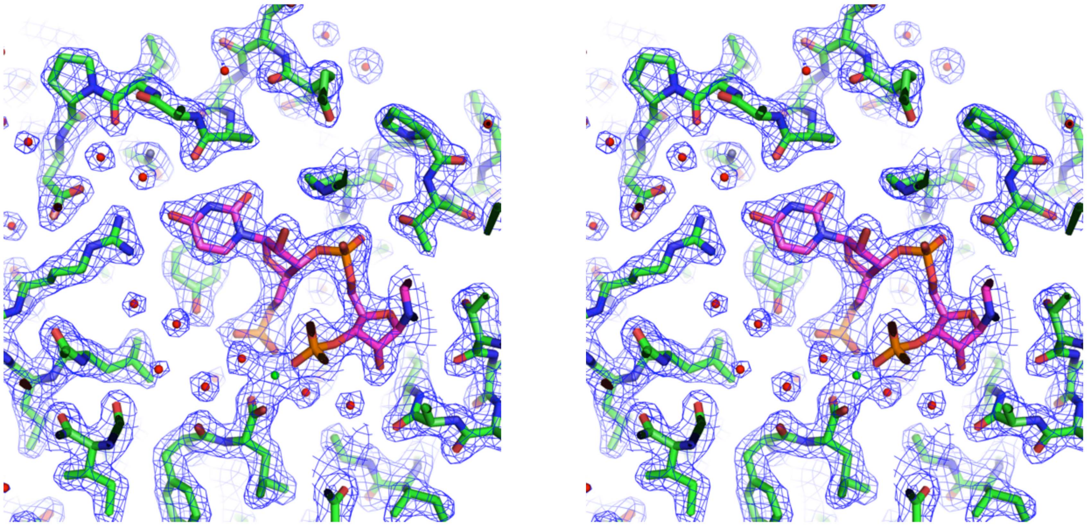

Supplementary Figure 10: Representative electron density map of the MID/PIWI interface in the *Rs*Ago-RNA/DNA complex. Representative stereo image of the 2*F*o - *F*c map contoured at 1.5 σ (blue mesh).

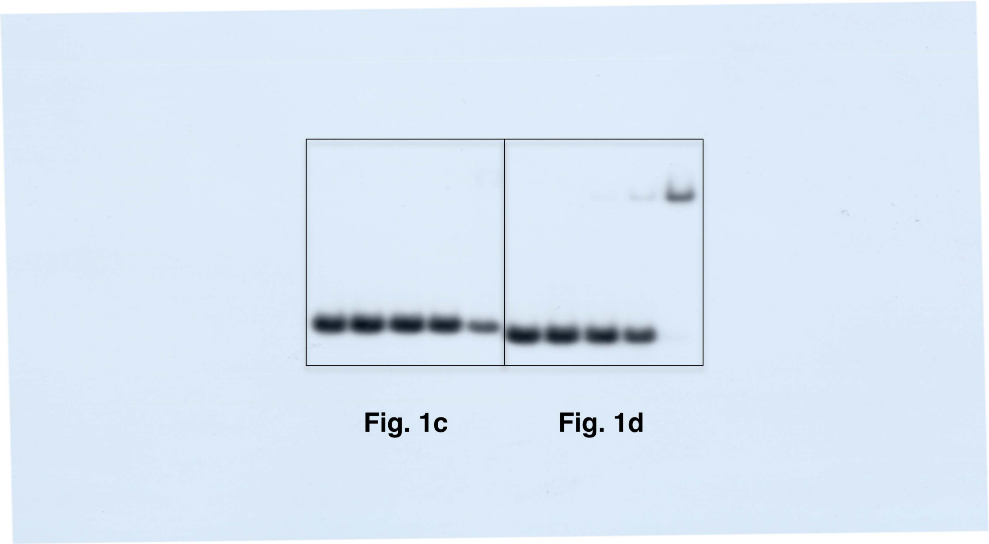

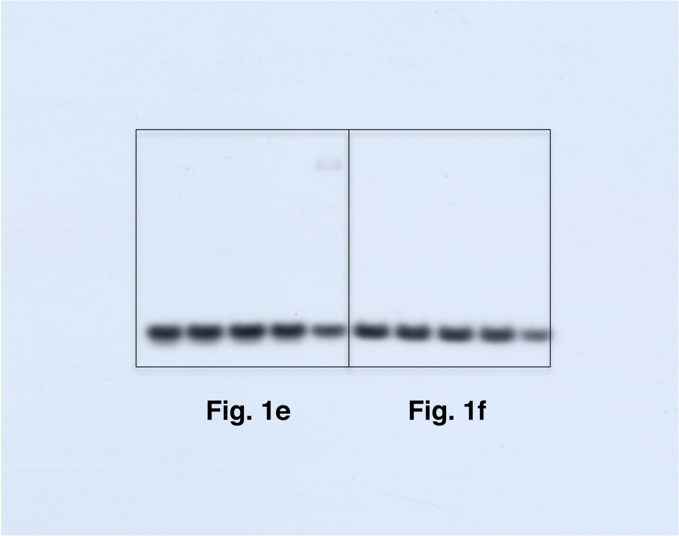

Supplementary Figure 11: **Uncropped scans of the electrophoresis mobility shift assays using *Rs*Ago, 18-base guide strand and 18-base target strand.**

Supplementary Table 1: Sequence of synthetic RNA and DNA strands.

<table>
<tr><td>5′P-RNA (5′U-RNA)</td><td>5′ p-UUACAACCUACUACCUCG 3′</td></tr>
<tr><td>5′P-DNA</td><td>5′ p-ttacaacctactacctcg 3′</td></tr>
<tr><td>5′OH-RNA</td><td>5′OH-UUACAACCUACUACCUCG 3′</td></tr>
<tr><td>5′A-RNA</td><td>5′ p-AUACAACCUACUACCUCG 3′</td></tr>
<tr><td>5′C-RNA</td><td>5′ p-CUACAACCUACUACCUCG 3′</td></tr>
<tr><td>5′G-RNA</td><td>5′ p-GUACAACCUACUACCUCG 3′</td></tr>
<tr><td>5′P-RNA/RNA</td><td>5′ p-UUACAACCUACUACCUCG 3′
||||||||||||||||||
3′ AAUGUUGGAUGAUGGAGC 5′</td></tr>
<tr><td>5′P-RNA/DNA</td><td>5′ p-UUACAACCUACUACCUCG 3′
||||||||||||||||||
3′ aatgttggatgatggagc 5′</td></tr>
<tr><td>5′P-DNA/RNA</td><td>5′ p-ttacaacctactacctcg 3′
||||||||||||||||||
3′ AAUGUUGGAUGAUGGAGC 5′</td></tr>
<tr><td>5′P-DNA/DNA</td><td>5′ p-ttacaacctactacctcg 3′
||||||||||||||||||
3′ aatgttggatgatggagc 5′</td></tr>
<tr><td>5′P-RNA/70-base target DNA</td><td>5′ p-UUACAACCUACUACCUCG 3′
||||||||||||||||||
3′ aattatgatgatatttaacactattaatgttggatgatggagcatatctttattattgaaattctaaaat-p 5′</td></tr>
</table>

The RNA and DNA strands are shown in upper case red letters and lower case blue letters, respectively. The phosphorylated 5′ end of each guide strand is denoted by the letter “p”.

Supplementary Table 2: Helical parameters of the RNA/DNA heteroduplex bound to *Rs*Ago.

Guide RNA	Puckering	Target DNA	Puckering
U2	C3′-endo	A2′	C2′-endo
A3	C3′-endo	T3′	C1′-exo
C4	C3′-endo	G4′	C2′-endo
A5	C3′-endo	T5′	C1′-exo
A6	C3′-endo	T6′	C2′-endo
C7	C3′-endo	G7′	O1′-endo
C8	C3′-endo	G8′	C1′-exo
U9	C3′-endo	A9′	C2′-endo
A10	C3′-endo	T10′	C1′-exo
C11	C3′-endo	G11′	O1′-endo
U12	C3′-endo	A12′	C2′-endo
A13	C3′-endo	T13′	O1′-endo
C14	C3′-endo	G14′	C2′-endo
C15	C3′-endo	G15′	O1′-endo
U16	C3′-endo	A16′	C2′-endo
C17	C3′-endo	G17′	C2′-endo
G18	C3′-endo	C18′	C2′-endo

	Base Pairs-Axis				*Intra-Base Pairs*					
Base	*Xdisp*	*Ydisp*	*Inclin*	*Tip*	*Shear*	*Stretch*	*Stagger*	*Buckle*	*Propeller*	*Opening*
2-2′	-3.02	-0.60	2.1	-3.1	-0.31	0.02	0.65	-13.3	-19.9	-2.7
3-3′	-2.01	-0.55	6.8	-1.5	-0.03	-0.16	0.23	-13.5	-13.3	5.3
4-4′	-3.00	-0.05	6.4	-8.2	-0.09	-0.02	0.10	-3.6	-15.5	0.7
5-5′	-2.31	0.00	10.5	-1.4	-0.08	-0.05	-0.04	-15.2	-12.1	6.8
6-6′	-2.52	0.06	13.1	-5.4	0.06	-0.18	-0.01	-6.2	-11.6	3.1
7-7′	-1.96	0.09	12.4	-3.5	0.06	-0.07	0.26	-3.5	-9.5	3.7
8-8′	-1.41	0.26	13.8	-9.0	0.10	-0.03	0.41	-11.8	-9.9	0.9
9-9′	-1.33	-0.23	6.0	-14.1	-0.10	-0.16	0.18	-1.5	-5.4	2.2
10-10′	-1.97	-0.74	-1.0	-6.9	-0.11	-0.07	-0.29	-3.8	-15.6	3.1
11-11′	-2.52	-0.52	-9.1	9.0	0.04	-0.10	-0.02	5.2	-15.2	2.7
12-12′	-1.73	0.03	-2.4	-21.4	0.05	-0.09	0.58	-20.4	-1.8	0.1
13-13′	-1.61	0.27	-0.2	-9.9	-0.22	-0.12	0.30	-5.9	-17.5	5.2
14-14′	-2.38	0.49	2.2	-5.6	0.09	-0.06	0.24	-7.3	-15.6	-0.6
15-15′	-1.70	0.64	7.0	0.5	-0.15	0.01	0.31	-6.4	-11.7	0.3
16-16′	-1.91	-0.05	12.4	-1.0	-0.04	-0.10	0.16	-10.3	-13.7	3.7
17-17′	-1.72	-0.12	16.3	-10.9	-0.20	-0.02	-0.24	-8.7	-2.5	1.4
18-18′	-1.98	-0.25	17.1	-9.1	-0.38	-0.32	-0.71	-0.4	-0.3	-0.3

	Inter-Base Pairs					
Step	*Shift*	*Slide*	*Rise*	*Tilt*	*Roll*	*Twist*
2-2′/3-3′	1.52	-1.14	3.30	5.6	2.4	32.7
3-3′/4-4′	-0.46	0.76	3.05	1.2	-3.3	35.4
4-4′/5-5′	0.98	-0.98	3.56	5.0	11.6	31.4
5-5′/6-6′	-0.02	-0.55	3.21	3.2	3.7	30.7
6-6′/7-7′	0.89	-0.83	3.39	-0.3	11.2	29.4
7-7′/8-8′	0.30	-0.77	3.49	4.9	7.8	35.0
8-8′/9-9′	0.01	-0.35	3.11	1.5	3.8	28.3
9-9′/10-10′	0.31	-0.65	3.34	5.8	9.3	24.0
10-10′/11-11′	0.26	-0.97	3.10	-2.4	8.0	32.7
11-11′/12-12′	2.03	-1.07	5.60	11.1	-40.0	23.5
12-12′/13-13′	0.74	-1.20	2.92	6.9	3.8	25.3
13-13′/14-14′	-0.95	-1.03	3.27	1.3	0.6	33.2
14-14′/15-15′	0.31	-0.57	3.20	1.0	9.1	33.2
15-15′/16-16′	0.01	-1.00	3.34	3.4	7.5	30.1
16-16′/17-17′	0.91	-0.50	3.25	8.0	1.9	33.9
17-17′/18-18′	0.78	-0.31	3.34	7.0	12.6	31.1

Supplementary Table 3: Synthetic RNA and DNA chimeric duplexes.

<table>
<tr><td>RR/DD (guide/target)
- Positive control -</td><td>1 18
5′ p-UUACAACCUACUACCUCG 3′
||||||||||||||||||
3′ aatgttggatgatggagc 5′
1′ 18′</td></tr>
<tr><td>RR/RD (guide/target)</td><td>5′ p-UUACAACCUACUACCUCG 3′
||||||||||||||||||
3′ AAUGUUGGAUGatggagc 5′</td></tr>
<tr><td>RR/DR (guide/target)</td><td>5′ p-UUACAACCUACUACCUCG 3′
||||||||||||||||||
3′ aatgttggatgAUGGAGC 5′</td></tr>
<tr><td>RD/DD (guide/target)</td><td>5′ p-UUACAACCUACtacctcg 3′
||||||||||||||||||
3′ aatgttggatgatggagc 5′</td></tr>
<tr><td>RD/RD (guide/target)</td><td>5′ p-UUACAACCUACtacctcg 3′
||||||||||||||||||
3′ AAUGUUGGAUGatggagc 5′</td></tr>
<tr><td>RD/DR (guide/target)</td><td>5′ p-UUACAACCUACtacctcg 3′
||||||||||||||||||
3′ aatgttggatgAUGGAGC 5′</td></tr>
</table>

The 18-base chimeric strands are shown. RNA sequences and DNA sequences are represented in upper case red letters and lower case blue letters, respectively. Phosphorylated 5′-ends are denoted by the letter “p”.

Supplementary Table 4: List of primers used in this study.

RsAgo WT construct:
- Forward 5′- GAGAGACATATGGCCCCAGTGCAGGCTGCCGATG -3′
- Reverse 5′- GAGAGACTCGAGTTATCATAGGAACCAGCGGCTCCACTTG -3′

ΔN20 mutant:
- Forward 5′- GTGCCGCGCGGCAGCCATATGCAGCTAGTCTCGAATGGCTTC -3′
- Reverse 5′- GAAGCCATTCGAGACTAGCTGCATATGGCTGCCGCGCGGCAC -3′

ΔP45-W63 mutant:
- Forward 5′- GATTGTTCGCGATCTGCCGGATTTTGACGGGAAGCTTTTTCACC -3′
- Reverse 5′- GGTGAAAAAGCTTCCCGTCAAAATCCGGCAGATCGCGAACAATC -3′

K49A/R52A mutant:
- Forward 5′- GCCGGATCCATCGAAAGTGGCGGAAGAGGCCACCCGGCTGATGGGGTATTGG -3′
- Reverse 5′- CCAATACCCCATCAGCCGGGTGGCCTCTTCCGCCACTTTCGATGGATCCGGC -3′

H62A mutant:
- Forward 5′- GGGTATTGGTTTGTTGCTTGGTTTGACGGGAAG -3′
- Reverse 5′- CTTCCCGTCAAACCAAGCAACAAACCAATACCC -3′

R97A mutant:
- Forward 5′- CATCCCTGGCTTCTAGCAGCCCGACTGGATGATGC -3′
- Reverse 5′- GCATCATCCAGTCGGGCTGCTAGAAGCCAGGGATG -3′

R151A mutant:
- Forward 5′- GTCATCCCCGCCTTCGCGTTGAG -3′
- Reverse 5′- CTCAACGCGAAGGCGGGGATGAC -3′

K157A mutant:
- Forward 5′- CTTCGCGTTGAGCCCGGCAATCTACGAACCAGTAG -3′
- Reverse 5′- CTACTGGTTCGTAGATTGCCGGGCTCAACGCGAAG -3′

R204A mutant:
- Forward 5′- CAGGCGGAAGGCACAGCCTGGTGAG -3′
- Reverse 5′- CACCAGGCTGTGCCTTCCGCCTGAC -3′

R209A mutant:
- Forward 5′- GCCTGGTGAGGCAGGATTGCTGGG -3′
- Reverse 5′- CCCAGCAATCCTGCCTCACCAGGC -3′

R204A/R209A mutant:
- Forward 5′- GTCAGGCGGAAGGCACAGCCTGGTGAGGCAGGATTGCTGGGC -3′
- Reverse 5′- GCCCAGCAATCCTGCCTCACCAGGCTGTGCCTTCCGCCTGAC -3′

E242A mutant:
- Forward 5′- CGCAAAGCTGGCAGGGTCAAAGG -3′
- Reverse 5′- CCTTTGACCCTGCCAGCTTTGCG -3′

K245A mutant:
- Forward 5′- CAAAGCTGGAAGGGTCAGCGGAGAATTTCACTAG -3′
- Reverse 5′- CTAGTGAAATTCTCCGCTGACCCTTCCAGCTTTG -3′

T249A mutant:
- Forward 5′- GTCAAAGGAGAATTTCGCTAGATGCCTGTCTGCATTG -3′
- Reverse 5′- CAATGCAGACAGGCATCTAGCGAAATTCTCCTTTGAC -3′

Y260A mutant:
- Forward 5′- CATTGTTGGGCCACAACGCCAAGAAGCTTTTGAACG -3′
- Reverse 5′- CGTTCAAAAGCTTCTTGGCGTTGTGGCCCAACAATG -3′

R275A mutant:
- Forward 5′- GGCAGGCTACGCCACGGGCCCAAG -3′
- Reverse 5′- CTTGGGCCCGTGGCGTAGCCTGCC -3′

Y329A mutant:
- Forward 5′- CAAGGTAGAAGCTGTGTTCGAC -3′
- Reverse 5′- GTCGAACACAGCTTCTACCTTG -3′

Y463A mutant:
- Forward 5′- CCGAAACCCAGCTATCCATACCAAG -3′
- Reverse 5′- CTTGGTATGGATAGCTGGGTTTCGG -3′

K467A mutant:

Forward 5′- TATCCATACCGCGTCCCTGCTAC -3′

Reverse 5′- GTAGCAGGGACGCGGTATGGATAT -3′

Y463A/K467A mutant:

Forward 5′- CCGAAACCCAGCTATCCATACCGCGTCCCTGCTACTGACGCTC -3′

Reverse 5′- GTAGCAGGGACGCGGTATGGATAGCTGGGTTTCGGTCGTCCGG -3′

Q478A mutant:

Forward 5′- CGTTCCAACGGCGCAAGTCAGAATG -3′

Reverse 5′- CATTCTGACTTGCGCCGTTGGAACG -3′

R481A mutant:

Forward 5′- GCAGCAAGTCGCAATGCCAACTGTTC -3′

Reverse 5′- GAACAGTTGGCATTGCGACTTGCTGC -3′

T484A mutant:

Forward 5′- CAGAATGCCAGCTGTTCTTCTAG -3′

Reverse 5′- CTAGAAGAACAGCTGGCATTCTG -3′

R481A/T484A mutant:

Forward 5′- CAGCAAGTCGCAATGCCAGCTGTTCTTCTAG -3′

Reverse 5′- CTAGAAGAACAGCTGGCATTGCGACTTGCTG -3′

K506A mutant:

Forward 5′- CGACCTATGCAGCGTTGAATGGCAC -3′

Reverse 5′- GTGCCATTCAACGCTGCATAGGTCG -3′

R537A mutant:

Forward 5′- CTTTCGGGCAGCGCGACAGAAAAGCGC -3′

Reverse 5′- GCGCTTTTCTGTCGCGCTGCCCGAAAG -3′

R541A mutant:

Forward 5′- CGGACAGAAAAGGCCCAGCGGTTTGTC -3′

Reverse 5′- GACAAACCGCTGGGCCTTTTCTGTCCG -3′

R543A mutant:

Forward 5′- GAAAAGCGCCAGGCGTTTGTCGGCATC -3′

Reverse 5′- GATGCCGACAAACGCCTGGCGCTTTTC -3′

R537A/R541A/R543A mutant:

Forward 5′- CTTTCGGGCAGCGCGACAGAAAAGGCCCAGGCGTTTGTCGGCATC -3′

Reverse 5′- GATGCCGACAAACGCCTGGGCCTTTTCTGTCGCGCTGCCCGAAAG -3′

ΔS536-R541 mutant:

Forward 5′- GGGGCTGGCGGAGCTTTCGGGCCAGCGGTTTGTCGGCATCACCAC -3′

Reverse 5′- GTGGTGATGCCGACAAACCGCTGGCCCGAAAGCTCCGCCAGCCCC -3′

R606A mutant:

Forward 5′- GTATTCCACGCCCACGCTCCACTGAAACGGGTG -3′

Reverse 5′- CACCCGTTTCAGTGGAGCGTGGGCGTGGAATAC -3′

S638A/H639A/D640A mutant:

Forward 5′- GTCACCGTCGCGGCTGCCCACCCATTCGTGCTCATCG -3′

Reverse 5′- CGAATGGGTGGGCAGCCGCGACGGTGACAAACGCCATCTG -3′

N686A/K692A mutant:

Forward 5′- GGTTGCCTCCCCGCAGTTGATCGCGAGGGCAAACACACCTTTGCC -3′

Reverse 5′- CTCGCGATCAACTGCGGGGAGGCAACCGCCAAAAGGCGTGTCAGC -3′

R731A mutant:

Forward 5′- GCCTTTCATGGGCCTCCACGCTTCCC -3′

Reverse 5′- GGGAAGCGTGGAGGCCCATGAAAGGC -3′

R754A mutant:

Forward 5′- GAGCTTTTGGGTGCTCTCAAAAGCATTCCC -3′

Reverse 5′- GGGAATGCTTTTGAGAGCACCCAAAAGCTC -3′

ΔL777 mutant:

Forward 5′- GGAGCCGCTGGTTCTAATAACTCGAGCACCAC -3′

Reverse 5′- GTGGTGCTCGAGTTATTAGAACCAGCGGCTCC -3′

Supplementary Notes

Widespread recognition of the heteroduplex by the PIWI domain and the mechanism of defective slicer activity of *Rs*Ago

The PIWI domain directly interacted with the heteroduplex through four main sites (Supplementary Fig. 6a). At the first site, a loop consisting of S534-R541 (hereafter termed loop L1, based on *Tt*Ago nomenclature) and the base region of loop L1 fitted into the minor groove of the heteroduplex and bound to both strands. The R537 and R543 side chains interacted with nucleotides 10 (2′-OH), 13 (2′-OH and O3′) and 14 (phosphate group) of the guide RNA strand, and the E532 main chain formed a hydrogen bond with nucleotide 9′ (phosphate group) of the target DNA strand (Supplementary Fig. 3d,e,i). In addition, the R541 side chain formed hydrogen bonds with nucleotide 11 (O2 and 2′-OH) of the guide RNA strand and nucleotides 9′ (O2) and 10′ (O4′) of the target DNA strand (Supplementary Fig. 3d,i). In *Tt*Ago, loop L1 functions as a gate for the catalytic glutamate residue to move into the active site[16]. In this context, loop L1 of *Rs*Ago was in a closed configuration. At the second site, a loop consisting of H605-K609 (hereafter loop L3) fitted into the minor groove of the heteroduplex and bound to both strands. On loop L3, the side chain R606 formed a hydrogen bond with nucleotides 13 (2′-OH) and 14 (O4′) of the guide RNA strand (Supplementary Fig. 3e). In addition, the side chain of K609 and the main chain of R610 interacted with the phosphate groups of nucleotides 15 and 16 of the guide RNA strand, respectively (Supplementary Fig. 3e). In *Tt*Ago, loop L3 also interacted with both the guide DNA and the target DNA/RNA strands in several conformational states[8,9,16]. At the third site, the side chains of N686 and K692 formed hydrogen bonds with nucleotides 6 (phosphate group) and 4 (2′-OH and O2) of the guide RNA strand, respectively (Supplementary Fig. 3b). In addition, the side chain of R731 interacted with the phosphate groups of nucleotides 3 and 4 of the guide RNA strand (Fig. 3a). Similar interactions via an arginine residue equivalent to R731 have also been observed in RNA-guided eukaryotic Argonaute proteins[10-14]. This residue is not conserved in DNA-guided prokaryotic Argonaute proteins (Supplementary Fig. 4) suggesting that the interaction via the arginine residue at the third site is a characteristic feature of RNA-guided Argonaute proteins. At the fourth site, the side chain of S638 and the main chains of H639 and D640 formed hydrogen bonds with the phosphate group of nucleotide 11′ of the target DNA strand (Supplementary Fig. 3j). In the

vicinity of the fourth site, the three catalytic residues DDD/H (D478, D546, and D660 in *Tt*Ago) were replaced by GHE (G529, H605, and E746) in *Rs*Ago (Supplementary Fig. 6b,c). Additionally, access to the active site by the E569-containing loop (corresponding to loop L2 of *Tt*Ago that contained the fourth catalytic residue E512), a conformational change to form a catalytic tetrad[12,16] was completely blocked by loop L1 (Supplementary Fig. 6b,c). These are likely the main causes of slicer-activity deficiency in *Rs*Ago.

Interactions and additional functions of the L1 and L2 linkers

Our structural data showed that the linker regions directly interacted with the guide and the target strands (Fig. 2c). At the L1 linker region, a long β-hairpin of the L1 linker interacted with the heteroduplex at two sites (Supplementary Fig. 7a). At the basal site that is close to the PAZ domain, the R151 side chain and the M176 and Y178 main chains formed hydrogen bonds with the phosphate groups of nucleotides 8 and 9 of the guide RNA strand (Supplementary Fig. 3c). At the front-end site, the K157 side chain formed a hydrogen bond with the phosphate group of nucleotide 13′ of the target DNA strand (Supplementary Fig. 3j). To clarify the functional roles of the interaction between the L1 linker and the heteroduplex, we performed a plasmid DNA silencing assay using R151A and K157A variants. The analysis showed that both the mutations significantly reduced DNA silencing activity compared to wild-type (Supplementary Fig. 7c). These results indicated that the interactions of R151 and K157 with the target strand were crucial for DNA silencing activity. On the other hand, the front-end site of the equivalent long β-hairpin is separate from the target strand in *Tt*Ago, unlike in *Rs*Ago (Supplementary Fig. 7a,b). This may be due to the presence of a negatively charged amino acid residue (D154) in *Tt*Ago at the equivalent position of K157. The interaction between K157 and the target DNA strand might be a specific feature of RNA-guided DNA-targeting Argonaute.

The L2 linker interacted with the duplex at two sites (Fig. 2c). At the first site, the helix α8-turn-helix α9 segment interacted with the seed region of the duplex (Supplementary Fig. 3h). On helix α8 of this segment, the K245 side chain formed hydrogen bonds with nucleotides 7 (2′-OH and O2) and 8 (O4′) of the guide RNA strand, and the T249 side chain formed a hydrogen bond with nucleotide 6′ (O3′) of the guide DNA strand (Supplementary Fig. 3d,h). On the other hand, on helix α9 of this segment, the Y260 side chain formed a hydrogen bond with nucleotide 5′ (phosphate group) of the target DNA strand, and the R275 side chain interacted with

nucleotides 7 (O3′) and 8 (phosphate group) of the guide RNA strand (Supplementary Fig. 3c,h). To evaluate the importance of these hydrogen bonds, we used a plasmid DNA silencing assay and found that substitution of K245 or R275 with alanine showed the reduction of the activity (Supplementary Fig. 7c). In *Hs*Ago2, it has been suggested that the fitting of helix α7, which corresponds to the helix α8-turn-helix α9 segment of *Rs*Ago, into the minor groove of the seed region is crucial for the target binding (Fig. 7a,b)[15]. Based on this view, we infer that the interaction of the helix α8-turn-helix α9 segment with the heteroduplex is important for the functional expression of *Rs*Ago. At the second site, the adenine of nucleotide 1′ of the target DNA strand penetrated a narrow pocket formed by the L2 linker (Y329, E340 and Y341) and the PIWI domain (R670, Q689 and L690), where the Y329 side chain of the L2 linker formed a hydrogen bond to the adenine N6 amine (Supplementary Fig. 3f,g). In a plasmid DNA silencing assay, we found that mutation of Y329 to alanine reduced the activity of *Rs*Ago (2.4-fold), suggesting the importance of this residue (Supplementary Fig. 7c).
